# Supplementary material for: A systematic review and meta-analysis of transdiagnostic cognitive behavioural therapies for emotional disorders
Source: Nat Hum Behav. 2024 Jan 16;8(3):493–509. doi: 10.1038/s41562-023-01787-3 (PMC10963275; doi:10.1038/s41562-023-01787-3)
Supplement: Supplementary file 1 — Supplementary Tables 1–8 and Figs. 1–27. [file 41562_2023_1787_MOESM1_ESM.pdf]

nature human behaviour

Article https://doi.org/10.1038/s41562-023-01787-3

A systematic review and meta-analysis of transdiagnostic cognitive behavioural therapies for emotional disorders

In the format provided by the authors and unedited

Supplementary Materials

"A systematic review and meta-analysis of transdiagnostic cognitive behavioral therapies for emotional disorders"

Supplementary Tables 4

Supplementary Table 1. Overview of content and setup of the investigated TD-CBT protocols 4

Supplementary Table 2. Search strings for all databases that were included in the systematic search 8

Supplementary Table 3. Overview of reasons for exclusion for each study that was full-text screened 9

Supplementary Table 4. Within-group effect sizes for TD treatments at pretest compared to the end of treatment and follow-up (FU) for depression and anxiety 18

Supplementary Table 5. Between-group effect sizes of depressive and anxiety symptoms for transdiagnostic treatments compared to control groups at the end of treatment, excluding outliers. 19

Supplementary Table 6. Between-group effect sizes of depressive and anxiety symptoms for transdiagnostic treatments compared to control groups at follow-up, excluding outliers. 20

Supplementary Table 7. Within-group effect sizes for TD treatments at pretest compared to the end of treatment and follow-up (FU) for depression and anxiety applying 0.3 as a correlation 22

Supplementary Table 8. Within-group effect sizes for TD treatments at pretest compared to the end of treatment and follow-up (FU) for depression and anxiety applying 0.7 as a correlation 23

Supplementary Figures 24

Supplementary Figure 1. Summary plot of risk of bias assessment 24

Supplementary Figure 2. Forest plot for depression comparing TD-CBT vs. controls at 3-month follow-up 24

Supplementary Figure 3. Forest plot for depression comparing TD-CBT vs. controls at 6-month follow-up 25

Supplementary Figure 4. Forest plot for depression comparing TD-CBT vs. controls at 12-month follow-up 25

Supplementary Figure 5. Forest plot for depression comparing TD-CBT vs. controls at 24-month follow-up 26

Supplementary Figure 6. Forest plot for anxiety comparing TD-CBT vs. controls at 3-month follow-up 26

Supplementary Figure 7. Forest plot for anxiety comparing TD-CBT vs. controls at 6-month follow-up 27

Supplementary Figure 8. Forest plot for anxiety comparing TD-CBT vs. controls at 12-month follow-up 27

Supplementary Figure 9. Forest plot for anxiety comparing TD-CBT vs. controls at 24-month follow-up 28

Supplementary Figure 10. Forest plot for depression from pre- to post-treatment 29

Supplementary Figure 11. Forest plot for depression from pre-treatment to 3-month follow-up 30

Supplementary Figure 12. Forest plot for depression from pre-treatment to 6-month follow-up 31

Supplementary Figure 13. Forest plot for depression from pre-treatment to 12-month follow-up 31

Supplementary Figure 14. Forest plot for depression from pre-treatment up to 24-month follow-up 32

Supplementary Figure 15. Forest plot for anxiety from pre- to post-treatment 33

Supplementary Figure 16. Forest plot for anxiety from pre-treatment to 3-month follow-up 34

Supplementary Figure 17. Forest plot for anxiety from pre-treatment to 6-month follow-up 35

Supplementary Figure 18. Forest plot for anxiety from pre-treatment to 12-month follow-up 35

Supplementary Figure 19. Forest plot for anxiety from pre-treatment up to 24-month follow-up 36

Supplementary Figure 20. Controlled Effects: Funnel plots for depression, a) without and b) with Trim and Fill Method 37

Supplementary Figure 21. Controlled Effects: Funnel plots for anxiety, a) without and b) with Trim and Fill Method 38

Additional Bayesian Analyses 39

1. **Statistical Procedure** 39

1.1 Forest Plots of Comparisons of TD-CBT and DS-CBT **40**

Supplementary Figure 22. Forest plot for depression comparing TD-CBT and DS-CBT (Bayesian analyses) 40

Supplementary Figure 23. Forest plot for anxiety comparing TD-CBT and DS-CBT (Bayesian analyses) 41

1.2 Sensitivity Analyses with Different Priors of Comparisons TD-CBT and DS-CBT **42**

Supplementary Figure 24. Forest plot for depression comparing TD-CBT and DS-CBT applying a non-informative uniform prior with a wide range and a Cauchy prior with a large scale 42

Supplementary Figure 25. Forest plot for anxiety comparing TD-CBT and DS-CBT applying a non-informative uniform prior with a wide range and a Cauchy prior with a large scale 43

Supplementary Figure 26. Forest plot for depression comparing TD-CBT and DS-CBT applying a weakly informative prior with a smaller distribution and a less conservative Half-Cauchy prior 44

Supplementary Figure 27. Forest plot for anxiety comparing TD-CBT and DS-CBT applying a weakly informative prior with a smaller distribution and a less conservative Half-Cauchy prior 45

Supplementary Tables

Supplementary Table 1. Overview of content and setup of the investigated TD-CBT protocols

					Treatment Components / Modules									
**Protocol**	**Trans-diagnostic core construct**	**Theoretical Basis**	**Number of sessions**	**Clinician level of training**	**Psycho-education**	**Motivation**	**Cognitive restructuring**	**Exposure**	**Behavioral activation**	**Relapse prevention**	**Mindfulness**	**Social skills**	**Others**	**Studies with Adaptations**
**Individual Setting**														
Unified Protocol (UP) (Barlow et al., 2011)	negative affect	CBT + emotion regulation	12-18	doctoral students, post-doc fellows, licensed therapists	x	x	x	x		x	x			- brief version in combination with the NICE guideline for common mental health disorders (Corpas et al., 2021)<br>- ESTEEM (Pachankis et al., 2022)
Transdiagnostic Behavior Therapy (TBT) (Gros, 2014)	avoidance	common treatment components / themes across CBT protocols	12	masters- or doctoral-level project therapists that received extensive training	x	x		x		x				
Emotion Regulation Therapy (ERT) (Mennin & Fresco, 2014)	emotion regulation	mechanism-targeted CBT integrating findings from affect science	20	doctoral students, post-doc fellows, licensed therapists	x		x	x		x	x			

Group Setting

Multimodal Intervention (MMI) (Ejeby et al., 2014)	not specified	CBT (mix of existing group interventions)	32 (150min) +2 individual	assistant nurses with brief training			x					x	- physical exercise	
False Safety Behavior Elimination Therapy (F-SET) (Schmidt et al., 2012)	false safety behavior	CBT + emotional processing model of fear	10 (120min)	master's level students, postdoc fellow with clinical experience	x			x	x				- reduction of safety behavior	
Anxiety Treatment Protocol (Norton & Hope, 2005)	not specified	CBT	12 (120min)	doctoral level students experienced in providing CBT	x		x	x		x				
UP group version (de Ornelas et al., 2017)	negative affect	CBT + emotion regulation	12 (120min)	n.a.	x	x	x	x		x	x	x	- relaxation	- brief version (Corpas et al. 2021)
Dialectical Behavior Therapy Skills Training (DBT-ST) (Neacsiu et al., 2014)	emotion dysregulation	CBT (DBT-focus)	16 (120min) +1 individual	clinical psychologist, social worker			x				x	x	- distress tolerance	
Culturally Adapted Cognitive Behavior Therapy Plus Problem Management (CA-CBT+) (Kananian et al., 2020)	distress	Culturally Adapted Cognitive Behavioral Therapy	12	Farsi-speaking therapists, trained in CA-CBT+	x	x					x	x	- problem solving -stretching exercises	
Group Treatment for Repetitive Negative Thinking	rumination	classical and operant learning, the role of	7	senior CBT-trained psychologist and senior psychiatrist	x		x		x					

(RNT-G) (Rogiers et al., 2022)		cognitions, attention shifting, Behavioral activation, Positive Psychology												
Affect Regulation Training (ART) (Berking & Whitley, 2014)	affect regulation	CBT with elements from other psychotherapeutic approaches that focus on affect regulation	7	psychologists with at least advanced clinical training	x					x	x			- intensive ART format with 180 min weekly sessions +1 booster session (Berking et al., 2019)
**Internet-based Setting**														
velibra (Berger et al., 2017)	not specified	CBT	6	(unguided intervention)	x		x	x	x	x	x	x		
Wellbeing course (Titov et al., 2011)	not specified	CBT	5	accredited therapists/ CBT-trained counsellors	x		x	x	x	x				
Anxiety Program (Titov et al., 2010)	not specified	CBT	8	clinical psychologist w/o postgrad training	x		x	x	x	x		x	- online discussion forum	
Worry and Sadness Program (Newby, 2013)	rumination	CBT	6	accredited psychotherapist/clinical psychologist	x		x	x	x	x			- optional additional material (sleep, assertiveness, positive psychology)	

UP, Internet-based version (Tulbure et al., 2018)	negative affect	CBT	9	specifically trained graduate students	x	x	x	x		x	x		- 12 modules of Internet-based UP plus DBT strategies (González-Robles et al., 2020)<br>- 12/16 modules of Internet-based UP plus DBT strategies (16 module version: additional positive affect regulation strategies) (Díaz-García et al., 2021)
Mindfulness-Enhanced iCBT (Kladnitski et al., 2020)	not specified	CBT, MBCT, MBSR	6	clinical psychologist	x		x	x	x	x	x	- optional additional material (sleep, assertiveness)	
Internet-based Mindfulness Intervention for Emotional Distress (iMIED) (Li et al., 2023)	mindfulness	integrates rationales and practices from UP and mindfulness-based interventions	8	internet self-help version	x		x	x			x		

Note. Several studies investigated a TD-CBT treatment that aggregated components and themes from several different CBT protocols but cannot be considered a stand-alone TD-CBT protocol (Erickson et al., 2007; Fernández-Rodríguez et al., 2022; Kunst et al., 2022; Roy-Byrne et al., 2010).

Supplementary Table 2. Search strings for all databases that were included in the systematic search

	**Search string**
**Pubmed & MEDLINE**	("transdiagnostic"[Text Word] OR "unified"[Text Word] OR "unspecific"[Text Word]) AND ("anxi*"[Text Word] OR "depress*"[Text Word] OR "emotional disorder*"[Text Word] OR "common mental health disorder*"[Text Word]) AND ("CBT"[Text Word] OR "cognitive behavioral therapy"[Text Word] OR "cognitive behavioural therapy"[Text Word] OR "cognitive behavior therapy"[Text Word] OR "cognitive behaviour therapy"[Text Word] OR "Internet-Based Intervention"[Text Word] OR "internet-based"[Text Word] OR "internetbased"[Text Word] OR "ICBT"[Text Word] OR "internet*"[Text Word] OR "web-based"[Text Word] OR "webbased"[Text Word] OR "individual"[Text Word] OR "group"[Text Word]) AND ("randomized controlled trial"[Text Word] OR "randomised controlled trial"[Text Word] OR "RCT"[Text Word] OR "control group"[Text Word] OR "control condition"[Text Word])
**PsycINFO**	TX((“transdiagnostic" OR "unified" OR "unspecific”) AND (”anxi*" OR "depress*" OR "emotional disorder*" OR "common mental health disorder*") AND (“CBT" OR "cognitive behavio#ral therapy" OR "cognitive behavio#r therapy" OR "Internet-Based Intervention" OR "internet?based" OR "ICBT" OR "internet*" OR "web?based" OR "individual" OR "group”) AND (“randomi?ed controlled trial" OR "RCT" OR "control group" OR "control condition”))
**Google Scholar**	-
**medRxiv (incl. bioRxiv)**	for term "transdiagnostic AND "common mental health disorder" AND "cognitive behavioral therapy" AND "randomized controlled trial"
**OSF Preprints**	(transdiagnostic OR unified OR unspecific) AND (anxi* OR depress* OR "emotional disorder*" OR "common mental health disorder*") AND (CBT OR "cognitive behavioral therapy" OR "cognitive behavioural therapy" OR "cognitive behavior therapy" OR "cognitive behaviour therapy" OR "internet-based intervention" OR internet-based OR "internet based" OR ICBT OR internet* OR web-based OR "web based" OR individual OR group) AND ("randomized controlled trial" OR "randomised controlled trial" OR RCT OR "control group" OR "control condition")

Supplementary Table 3. Overview of reasons for exclusion for each study that was full-text screened

	**Reason for exclusion**											
	secondary analysis	no clinician-established diagnosis	different primary diagnosis	not based on trans-diagnostic CBT principles	tailored treatment	outcome measure of anxiety or depression not available	no RCT	study protocol	meta-analysis	factual text	other	comment
Aghighi et al. (2022)						x						
Aguilera-Martín et al. (2022)								x				
Alatiq (2021)							x					
Allen et al. (2009)	x											
Andersen et al. (2016)									x			
Applebaum et al. (2022)		x										
Arai et al. (2022)							x					
Arani et al. (2022)							x					
Arch et al. (2012)				x								ACT is compared to traditional CBT
Arch et al. (2013)				x								intervention group: mindfulness-based stress reduction, com-parison was CBT - but not transdiagnostic

Arnfred et al. (2017)								x				
Batterham et al. (2021)		x										
Bautista et al. (2021)							x					
Bentley et al. (2017)		x										Self-Injurious Thoughts and Behaviors Inter-view–Self-Report (SITBI-SR) is used for diagnosis
Berger et al. (2014)					x							
Berking et al. (2022)			x									Binge Eating Disorder
Black et al. (2023)					x							
Bogdanov et al. (2021)		x										
Bolton et al. (2014)		x										
Brenes et al. (2012)				x								telephone-delivered CBT
Bressi et al. (2010)				x								intervention group gets short-term psychody-namic psychotherapy (STPP)
Buckner et al. (2019)			x									main target: Cannabis use disorder (CUD)
Bullis et al. (2014)							x					
Cano-Vindel et al. (2021)		x										
Cano-Vindel et al. (2022)	x											

Carlbring et al. (2001)				x								
Carlbring et al. (2011)					x							
Cassiello-Robbins et al. (2020)											no control group	
Conklin et al. (2021)	x											
Craske et al. (2007)					x							
Craske et al. (2009)					x							
Craske et al. (2011)					x							
De Fabritiis et al. (2022)								x				
de Ornelas Maia et al. (2015)							x					
De Paul & Caver (2021)		x										reconfirmed with the authors, they did not use an interview
Dear et al. (2011)							x					
Dear et al. (2018)		x										
Dear et al. (2022)		x										
DeTore et al. (2022)		x										
Dirkse et al. (2020)											no control group	
Dong et al. (2022)				x								
Durand et al. (2022)	x											

Ebert et al. (2013)		x										
Ebert et al. (2013)	x											
Ejeby et al. (2014)						x						
Ejeby (2022)											dissertation, one of the studies already included	
Ekkers et al. (2011)		x										
Falahat et al. (2022)		x										
Fortuna et al. (2020)		x										
Francis et al. (2022)				x								
García-Escalera et al. (2017)									x			
González-Blanch et al. (2021)	x											
González-Blanch et al. (2023)	x											
González-Robles et al. (2021)	x											
González-Robles et al. (2022)	x											12-month follow-up values were included in our meta-analysis
Gros (2014)							x					
Gros (2015)								x				
Gros & Coyne (2022)	x											
Gros & Oglesby (2019)										x		

Gros et al. (2016)										x		
Gros et al. (2017)							x					
Gros et al. (2019)							x					
Guliani et al. (2022)	x											
Gulliver et al. (2021)		x										
Hadjistavropoulos et al. (2020)		x										
Hadjistavropoulos, Thiessen et al. (2020)		x										
Hagberg et al. (2023)		x										
Harrer et al. (2021)				x								
Hoiles et al. (2022)		x										
Hvenegaard et al. (2019)											focused on depres-sion, not in line with “broadspec-trum” CBT ap-proach of unified TD treatments	
Ito et al. (2016)							x					
Johansson et al. (2012)					x							
Johnson et al. (2017)											not in line with “broadspectrum” CBT approach of unified TD treat-ments	

Karyotaki et al. (2022)		x										
Kim et al. (2009)											intervention group: mindfulness-based CT	
Knefel et al. (2022)		x										
Korrelboom et al. (2014)				x								
Krieger et al. (2019)				x								
Kristjánsdóttir et al. (2019)							x					
Laposa et al. (2017)							x					
Maia et al. (2013)							x					
Marks et al. (2004)				x								
Mason et al. (2022)				x								
Mohammadi et al. (2018)			x									main target: Borderline Personality Disorder (BPD)
Moritz et al. (2020)				x								
Morris et al. (2023)		x										
Muschalla et al. (2020)				x								
Newby et al. (2017)							x					
Niveau et al. (2022)		x										

Niemeijer et al. (2023	x											
Nordgren et al. (2014)					x							
O'Donnell et al. (2021)			x									main target: PTSD
Olthuis et al. (2014)				x								
Pachankis et al. (2020)		x										
Papini et al. (2022)		x										
Patel et al. (2003)				x								
Paulus et al. (2020)		x										
Peláez et al. (2022)								x				
Peros et al. (2022)		x										
Peynenburg et al. (2022)		x										
Pittig et al. (2021)											includes under-age participants	
Reinholt et al. (2017)							x					
Riccardi et al. (2017)							x					
Robichaud et al. (2020)		x										
Ruiz-Rodríguez et al. (2018)								x				
Sauer-Zavala et al. (2020)	x											

Sauer-Zavala et al. (2021)	x											
Sauer-Zavala et al. (2022)					x							
Schaeuffele et al. (2022)	x											
Schneider et al. (2020)		x										
Schulz-Heik et al. (2022)				x								
Senger et al. (2022)		x										
Sharma et al. (2022)		x										
Smith et al. (2022								x				
Soleimanian-Boroujeni et al. (2022)		x										
Soucy et al. (2021)		x										
Southward et al. (2022)	x											
Steele et al. (2018)	x											
Suchan et al. (2022)		x										
Sullivan et al. (2007)								x				
Thompson-Brenner et al. (2019)							x					preliminary pre-post evaluation
Titov et al. (2009)	x											

Uwadiale (2022)		x										
Vøllestad et al. (2011)											mindfulness-based stress reduction	
Waite et al. (2012)											focus on low self-esteem, not in line with “broadspec-trum” CBT ap-proach of unified TD treatments	
Weinzimmer et al. (2022)							x					
Weisberg et al. (2022)				x								
Weiss et al. (2015)		x										main target: PTSD
Wetherell et al. (2009)				x								
Wilner et al. (2020)	x											
Woods et al. (2020)	x											
Wuthrich & Rapee (2013)											inclusion criteron: ≥ 60 years	
Wuthrich et al. (2016)											inclusion criterion: ≥ 60 years	
Zabel et al. (2022)											focus on low self-esteem, not in line with “broadspec-trum” CBT ap-proach of unified TD treatments	

Supplementary Table 4. Within-group effect sizes for TD treatments at pretest compared to the end of treatment and follow-up (FU) for depression and anxiety

		Depression								Anxiety							
	Setting	*k*	d_{SMC}	*p*	*LL*	*UL*	I^2	*Q*	*p*	*k*	d_{SMC}	*p*	*LL*	*UL*	I^2	*Q*	*p*
Pre to Post-Treatment	All	52	1.14	<0.001	1.01	1.28	84.35	284.5	<0.001	52	1.08	<0.001	0.94	1.22	87.88	300.79	<0.001
	Individual	14	1.11	<0.001	0.88	1.34	58.65	35.92	<0.001	14	1.20	<0.001	0.92	1.49	72.86	40.45	<0.001
	Group	19	1.18	<0.001	0.87	1.49	94.49	91.83	<0.001	19	1.25	<0.001	0.79	1.70	97.52	143.77	<0.001
	Internet-based	19	1.19	<0.001	1.00	1.38	80.79	113.08	<0.001	19	1.00	<0.001	0.85	1.15	76.69	95.08	<0.001
Pre to 3-month FU	All	28	1.38	<0.001	1.12	1.64	92.42	227.58	<0.001	26	1.35	<0.001	1.08	1.62	93.45	194.62	<0.001
	Individual	6	1.47	<0.001	0.98	1.97	74.44	17.36	0.004	5	1.67	<0.001	0.99	2.35	82.96	18.71	<0.001
	Group	10	1.33	<0.001	0.71	1.95	97.20	55.30	<0.001	9	1.65	<0.001	0.69	2.61	98.72	85.91	<0.001
	Internet-based	12	1.42	<0.001	1.08	1.76	89.66	113.86	<0.001	12	1.19	<0.001	0.95	1.43	83.00	75.45	<0.001
Pre to 6-month FU	All	18	0.85	<0.001	0.75	0.94	32.62	22.98	0.150	19	0.91	<0.001	0.78	1.05	71.30	54.01	<0.001
	Individual	8	0.80	<0.001	0.63	0.98	45.70	11.33	0.125	8	0.92	<0.001	0.75	1.10	43.12	12.88	0.075
	Group	8	0.89	<0.001	0.78	1.00	0.05	7.18	0.410	9	0.88	<0.001	0.66	1.09	80.88	38.83	<0.001
	Internet-based	-	-	-	-	-	-	-	-	-	-	-	-	-	-	-	-
Pre to 12-month FU	All	10	1.36	<0.001	1.01	1.72	93.13	118.86	<0.001	9	1.19	<0.001	0.91	1.48	91.00	75.94	<0.001
	Individual	-	-	-	-	-	-	-	-	-	-	-	-	-	-	-	-
	Group	3	1.03	<0.001	0.53	1.53	75.08	8.34	0.015	-	-	-	-	-	-	-	-
	Internet-based	5	1.76	<0.001	1.31	2.20	88.57	35.71	<0.001	5	1.37	<0.001	0.89	1.84	92.07	45.40	<0.001
Pre to 24-month FU	All	5	1.75	<0.001	1.29	2.21	93.07	91.78	<0.001	5	1.47	<0.001	1.04	1.90	93.75	74.50	<0.001
	Individual	-	-	-	-	-	-	-	-	-	-	-	-	-	-	-	-
	Group	-	-	-	-	-	-	-	-	-	-	-	-	-	-	-	-
	Internet-based	4	1.96	<0.001	1.63	2.29	72.87	11.84	0.008	4	1.62	<0.001	1.21	2.03	86.32	19.49	<0.001

Supplementary Table 5. Between-group effect sizes of depressive and anxiety symptoms for transdiagnostic treatments compared to control groups at the end of treatment, excluding outliers.

		Depression								Anxiety							
		k	*g*	*p*	*LL*	*UL*	I^2	*Q*	*p*	*k*	*g*	*p*	*LL*	*UL*	I^2	*Q*	*p*
TD-CBT (all treatment formats) vs.	control	49	0.63	<0.001	0.53	0.73	49.82	96.71	<0.001	39	0.71	<0.001	0.61	0.81	33.80	57.41	0.022
	DS	9	0.09	0.269	-0.07	0.25	53.96	17.82	0.023	8	0.08	0.145	-0.03	0.18	3.79	5.38	0.613
	TAU	16	0.74	<0.001	0.58	0.9	48.48	28.07	0.021	13	0.71	<0.001	0.53	0.88	47.85	22.14	0.036
	other	12	0.24	0.002	0.09	0.39	0	6.50	0.838	13	0.14	0.128	-0.04	0.31	17.67	15.33	0.224
	WL	18	1.00	<0.001	0.81	1.20	54.03	37.55	0.003	17	0.85	<0.001	0.73	0.97	0.86	20.62	0.194
Individual TD-CBT vs.	control	16	0.88	<0.001	0.58	1.18	63.69	39.85	<0.001	12	1.08	<0.001	0.77	1.38	49.78	21.31	0.030
	DS	-	-	-	-	-	-	-	-	-	-	-	-	-	-	-	-
	TAU	4	1.08	<0.001	0.73	1.43	0	3.11	0.374	3	1.33	0.002	0.49	2.16	74.24	7.23	0.027
	other	5	0.49	0.011	0.11	0.86	25.73	5.99	0.200	5	0.25	0.187	-0.12	0.62	26.40	5.28	0.259
	WL	7	1.40	<0.001	0.87	1.93	71.24	23.89	<0.001	6	1.38	<0.001	0.74	2.02	77.84	18.05	0.003
Group-based TD-CBT vs.	control	19	0.52	<0.001	0.37	0.67	57.61	42.14	0.001	15	0.47	<0.001	0.34	0.61	33.84	19.09	0.162
	DS	3	0.16	0.306	-0.15	0.48	59.79	4.98	0.083	3	0.14	0.331	-0.14	0.41	49.59	3.69	0.158
	TAU	7	0.75	<0.001	0.43	1.07	75.36	18.03	0.006	7	0.72	<0.001	0.34	1.10	83.27	25.84	<0.001
	other	6	0.23	0.017	0.04	0.41	0.01	5.50	0.358	6	0.07	0.669	-0.24	0.37	49.27	9.49	0.091
	WL	6	1.14	0.048	0.01	2.27	95.76	27.50	<0.001	6	1.52	0.033	0.12	2.92	96.85	66.10	<0.001
Internet-based TD-CBT vs.	control	17	0.69	<0.001	0.54	0.84	52.20	34.10	0.005	15	0.66	<0.001	0.51	0.82	43.10	25.26	0.032
	DS	4	0.08	0.563	-0.18	0.33	70.37	11.37	0.010	4	0.03	0.662	-0.11	0.17	0	0.47	0.925
	TAU	6	0.79	<0.001	0.46	1.12	69.03	15.73	0.008	6	0.76	<0.001	0.43	1.09	69.15	16.09	0.007
	other	-	-	-	-	-	-	-	-	-	-	-	-	-	-	-	-
	WL	9	0.86	<0.001	0.72	1.01	0	7.89	0.444	9	0.83	<0.001	0.66	1.00	20.87	11.07	0.198

Supplementary Table 6. Between-group effect sizes of depressive and anxiety symptoms for transdiagnostic treatments compared to control groups at follow-up, excluding outliers.

			Depression								Anxiety							
			k	*g*	*p*	*LL*	*UL*	I^2	*Q*	*p*	*k*	*g*	*p*	*LL*	*UL*	I^2	*Q*	*p*
3-month FU	TD-CBT (all treatment formats) vs.	control	21	0.34	<0.001	0.23	0.45	33.57	30.02	0.069	20	0.25	0.002	0.10	0.41	65.48	51.69	<0.001
		DS-CBT	5	0.11	0.376	-0.14	0.37	77.81	15.73	0.003	5	-0.01	0.912	-0.18	0.16	52.62	8.48	0.075
		TAU	6	0.42	<0.001	0.26	0.59	6.45	6.77	0.238	4	0.49	<0.001	0.22	0.76	51.43	6.23	0.101
		other	9	0.23	0.019	0.04	0.42	20.26	9.11	0.333	8	0.24	0.009	0.06	0.42	0	9.69	0.207
		WL	6	1.23	<0.001	0.52	1.94	88.67	35.76	<0.001	5	1.16	0.014	0.23	2.09	90.47	39.86	<0.001
	Individual TD-CBT vs.	control	7	1.12	<0.001	0.48	1.77	86.76	32.19	<0.001	4	2.3	<0.001	1.27	3.33	77.67	12.3	0.006
	Group-based TD-CBT vs.	control	11	0.28	<0.001	0.14	0.42	22.82	15.95	0.101	10	0.42	<0.001	0.20	0.65	61.8	22.45	0.008
	Internet-based TD-CBT vs.	control	9	0.18	0.126	-0.05	0.42	73.17	27.64	<0.001	9	-0.05	0.409	-0.17	0.07	2.55	7.89	0.444
6-month FU	TD-CBT (all treatment formats) vs.	control	16	0.19	<0.001	0.09	0.28	11.22	18.92	0.217	18	0.21	<0.001	0.1	0.32	31.92	24.72	0.101
		DS-CBT	3	-0.01	0.937	-0.23	0.21	39.89	3.28	0.194	3	0.04	0.650	-0.15	0.24	22.99	2.33	0.312
		TAU	4	0.26	<0.001	0.15	0.38	0	0.74	0.864	4	0.32	0.001	0.13	0.52	47.22	5.67	0.129
		other	7	0.15	0.162	-0.06	0.37	10.92	6.13	0.409	8	0.19	0.050	0	0.38	0	6.19	0.518
		WL	3	0.63	0.019	0.1	1.15	47.71	3.82	0.148	4	0.62	0.065	-0.04	1.27	77.44	11.9	0.008
	Individual TD-CBT vs.	control	9	0.24	0.012	0.05	0.43	44.45	14.07	0.080	9	0.26	<0.001	0.15	0.36	0.01	9.4	0.309
	Group-based TD-CBT vs.	control	8	0.16	0.033	0.01	0.31	16.49	9.45	0.222	9	0.17	0.065	-0.01	0.36	46.53	14.41	0.072
	Internet-based TD-CBT vs.	control	-	-	-	-	-	-	-	-	-	-	-	-	-	-	-	-
12-month FU	TD-CBT (all treatment formats) vs.	control	10	0.30	<0.001	0.22	0.39	0	3.71	0.930	11	0.22	<0.001	0.12	0.32	24.83	10.52	0.396
		DS-CBT	4	0.13	0.247	-0.09	0.36	58.91	7.48	0.058	4	0.08	0.253	-0.06	0.23	0	0.87	0.832
		TAU	4	0.35	<0.001	0.24	0.47	0	0.30	0.960	4	0.36	<0.001	0.24	0.48	0	0.70	0.872
		other	3	0.23	0.030	0.02	0.44	0	0.03	0.985	3	0.21	0.044	0.01	0.42	0	0.10	0.950
		WL	-	-	-	-	-	-	-	-	-	-	-	-	-	-	-	-
	Individual TD-CBT vs.	control	3	0.33	<0.001	0.21	0.45	0	0.58	0.748	3	0.34	<0.001	0.20	0.47	9.71	1.67	0.434

	Group-based TD-CBT vs.	control	3	0.30	0.006	0.09	0.50	0	0.59	0.744	3	0.27	0.012	0.06	0.48	0	0.24	0.888
	Internet-based TD-CBT vs.	control	5	0.17	0.098	-0.03	0.38	54.63	8.93	0.063	5	0.11	0.116	-0.03	0.24	0	1.95	0.746
24-month FU	TD-CBT (all treatment formats) vs.	control	5	0.20	0.111	-0.05	0.46	80.63	16.44	0.002	5	0.14	0.092	-0.02	0.31	56.46	8.63	0.071
		DS-CBT	4	0.20	0.259	-0.14	0.54	81.90	16.15	0.001	4	0.11	0.344	-0.12	0.35	62.33	8.25	0.041
		TAU	-	-	-	-	-	-	-	-	-	-	-	-	-	-	-	-
		other	-	-	-	-	-	-	-	-	-	-	-	-	-	-	-	-
		WL	-	-	-	-	-	-	-	-	-	-	-	-	-	-	-	-
	Individual TD-CBT vs.	control	-	-	-	-	-	-	-	-	-	-	-	-	-	-	-	-
	Group-based TD-CBT vs.	control	-	-	-	-	-	-	-	-	-	-	-	-	-	-	-	-
	Internet-based TD-CBT vs.	control	4	0.20	0.259	-0.14	0.54	81.9	16.15	0.001	4	0.11	0.344	-0.12	0.35	62.33	8.25	0.041

Supplementary Table 7. Within-group effect sizes for TD treatments at pretest compared to the end of treatment and follow-up (FU) for depression and anxiety applying 0.3 as a correlation

		Depression								Anxiety							
	TD-CBT Setting	*k*	d_{SMC}	*p*	*LL*	*UL*	I^2	*Q*	*p*	*k*	d_{SMC}	*p*	*LL*	*UL*	I^2	*Q*	*p*
Pre to Post-Treatment	All	52	1.14	<0.001	1.01	1.27	83.75	279.75	<0.001	52	1.07	<0.001	0.93	1.21	87.25	296.21	<0.001
	Individual	14	1.11	<0.001	0.87	1.34	57.60	35.24	<0.001	14	1.20	<0.001	0.91	1.49	72.30	39.86	<0.001
	Group	19	1.17	<0.001	0.86	1.47	94.14	87.52	<0.001	19	1.24	<0.001	0.79	1.70	97.46	140.17	<0.001
	Internet-based	19	1.19	<0.001	1.00	1.38	80.75	112.91	<0.001	19	1.00	<0.001	0.85	1.15	76.57	94.69	<0.001
Pre to 3-month FU	All	28	1.38	<0.001	1.12	1.64	92.26	225.81	<0.001	26	1.35	<0.001	1.08	1.62	93.29	193.19	<0.001
	Individual	6	1.48	<0.001	0.98	1.98	73.42	17.34	0.004	5	1.67	<0.001	0.99	2.36	81.89	18.71	<0.001
	Group	10	1.33	<0.001	0.71	1.95	97.10	53.81	<0.001	9	1.65	<0.001	0.69	2.60	98.71	84.36	<0.001
	Internet-based	12	1.42	<0.001	1.08	1.76	89.66	113.86	<0.001	12	1.19	<0.001	0.95	1.43	83.00	75.45	<0.001
Pre to 6-month FU	All	18	0.84	<0.001	0.75	0.94	32.56	22.84	0.155	19	0.91	<0.001	0.78	1.05	70.32	53.59	<0.001
	Individual	8	0.80	<0.001	0.62	0.98	44.43	11.02	0.138	8	0.93	<0.001	0.75	1.10	41.73	12.41	0.088
	Group	8	0.89	<0.001	0.78	1.00	9.39	7.18	0.410	9	0.88	<0.001	0.66	1.09	80.88	38.83	<0.001
	Internet-based	-	-	-	-	-	-	-	-	-	-	-	-	-	-	-	-
Pre to 12-month FU	All	10	1.37	<0.001	1.02	1.72	92.48	112.31	<0.001	9	1.19	<0.001	0.91	1.48	90.20	70.54	<0.001
	Individual	-	-	-	-	-	-	-	-	-	-	-	-	-	-	-	-
	Group	3	1.05	<0.001	0.56	1.54	71.60	7.16	0.028	-	-	-	-	-	-	-	-
	Internet-based	5	1.76	<0.001	1.32	2.20	87.58	29.84	<0.001	5	1.37	<0.001	0.90	1.84	91.44	38.55	<0.001
Pre to 24-month FU	All	5	1.75	<0.001	1.29	2.21	92.76	85.53	<0.001	5	1.47	<0.001	1.04	1.90	93.44	69.57	<0.001
	Individual	-	-	-	-	-	-	-	-	-	-	-	-	-	-	-	-
	Group	-	-	-	-	-	-	-	-	-	-	-	-	-	-	-	-
	Internet-based	4	1.96	<0.001	1.63	2.29	72.87	11.84	0.008	4	1.62	<0.001	1.21	2.03	86.32	19.49	<0.001

Supplementary Table 8. Within-group effect sizes for TD treatments at pretest compared to the end of treatment and follow-up (FU) for depression and anxiety applying 0.7 as a correlation

		Depression								Anxiety							
	TD-CBT Setting	*k*	d_{SMC}	*p*	*LL*	*UL*	I^2	*Q*	*p*	*k*	d_{SMC}	*p*	*LL*	*UL*	I^2	*Q*	*p*
Pre to Post-Treatment	All	52	1.15	<0.001	1.02	1.28	85.10	290.68	<0.001	52	1.09	<0.001	0.94	1.23	88.54	306.39	<0.001
	Individual	14	1.11	<0.001	0.88	1.35	60.17	36.83	<0.001	14	1.20	<0.001	0.92	1.49	73.68	41.31	<0.001
	Group	19	1.18	<0.001	0.87	1.50	94.87	97.21	<0.001	19	1.25	<0.001	0.79	1.71	97.58	147.90	<0.001
	Internet-based	19	1.19	<0.001	1.01	1.38	80.86	113.33	<0.001	19	1.00	<0.001	0.85	1.16	76.87	95.68	<0.001
Pre to 3-month FU	All	28	1.38	<0.001	1.12	1.64	92.66	229.96	<0.001	26	1.35	<0.001	1.08	1.63	93.62	196.25	<0.001
	Individual	6	1.47	<0.001	0.98	1.96	75.83	17.38	0.004	5	1.67	<0.001	0.99	2.34	84.33	18.72	<0.001
	Group	10	1.33	<0.001	0.71	1.96	97.34	57.01	<0.001	9	1.65	<0.001	0.69	2.61	98.73	87.65	<0.001
	Internet-based	12	1.42	<0.001	1.08	1.76	89.66	113.86	<0.001	12	1.19	<0.001	0.95	1.43	83.00	75.45	<0.001
Pre to 6-month FU	All	18	0.85	<0.001	0.76	0.94	32.78	23.21	0.143	19	0.91	<0.001	0.78	1.05	72.71	54.67	<0.001
	Individual	8	0.81	<0.001	0.64	0.98	47.71	11.75	0.109	8	0.92	<0.001	0.76	1.09	45.42	13.55	0.060
	Group	8	0.89	<0.001	0.78	1.00	9.39	7.18	0.410	9	0.88	<0.001	0.66	1.09	80.88	38.83	<0.001
	Internet-based	-	-	-	-	-	-	-	-	-	-	-	-	-	-	-	-
Pre to 12-month FU	All	10	1.36	<0.001	1.00	1.71	93.95	127.69	<0.001	9	1.19	<0.001	0.90	1.48	92.01	84.00	<0.001
	Individual	-	-	-	-	-	-	-	-	-	-	-	-	-	-	-	-
	Group	3	1.01	<0.001	0.51	1.52	79.15	10.43	0.005	-	-	-	-	-	-	-	-
	Internet-based	5	1.75	<0.001	1.30	2.20	89.69	45.06	<0.001	5	1.36	<0.001	0.88	1.84	92.90	57.54	<0.001
Pre to 24-month FU	All	5	1.75	<0.001	1.29	2.21	93.40	99.20	<0.001	5	1.47	<0.001	1.04	1.90	94.08	80.49	<0.001
	Individual	-	-	-	-	-	-	-	-	-	-	-	-	-	-	-	-
	Group	-	-	-	-	-	-	-	-	-	-	-	-	-	-	-	-
	Internet-based	4	1.96	<0.001	1.63	2.29	72.87	11.84	0.008	4	1.62	<0.001	1.21	2.03	86.32	19.49	<0.001

Supplementary Figures

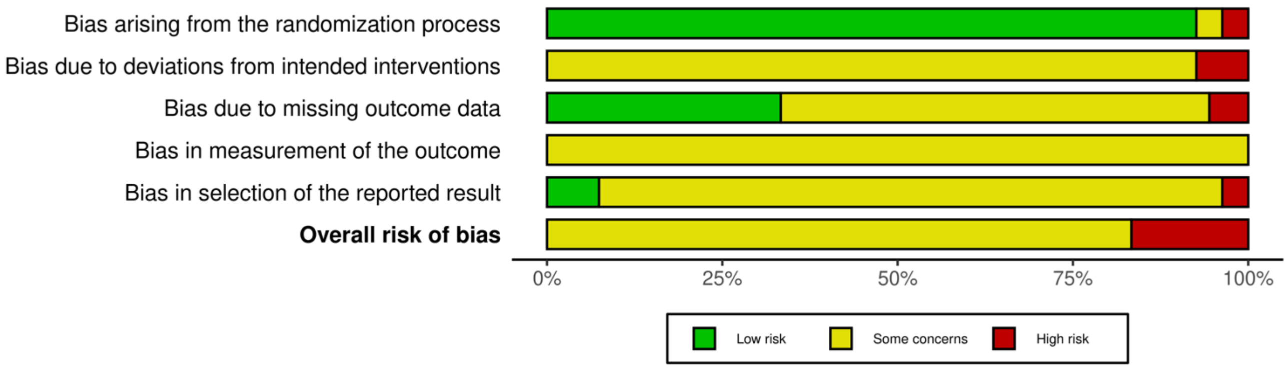

Supplementary Figure 1. Summary plot of risk of bias assessment

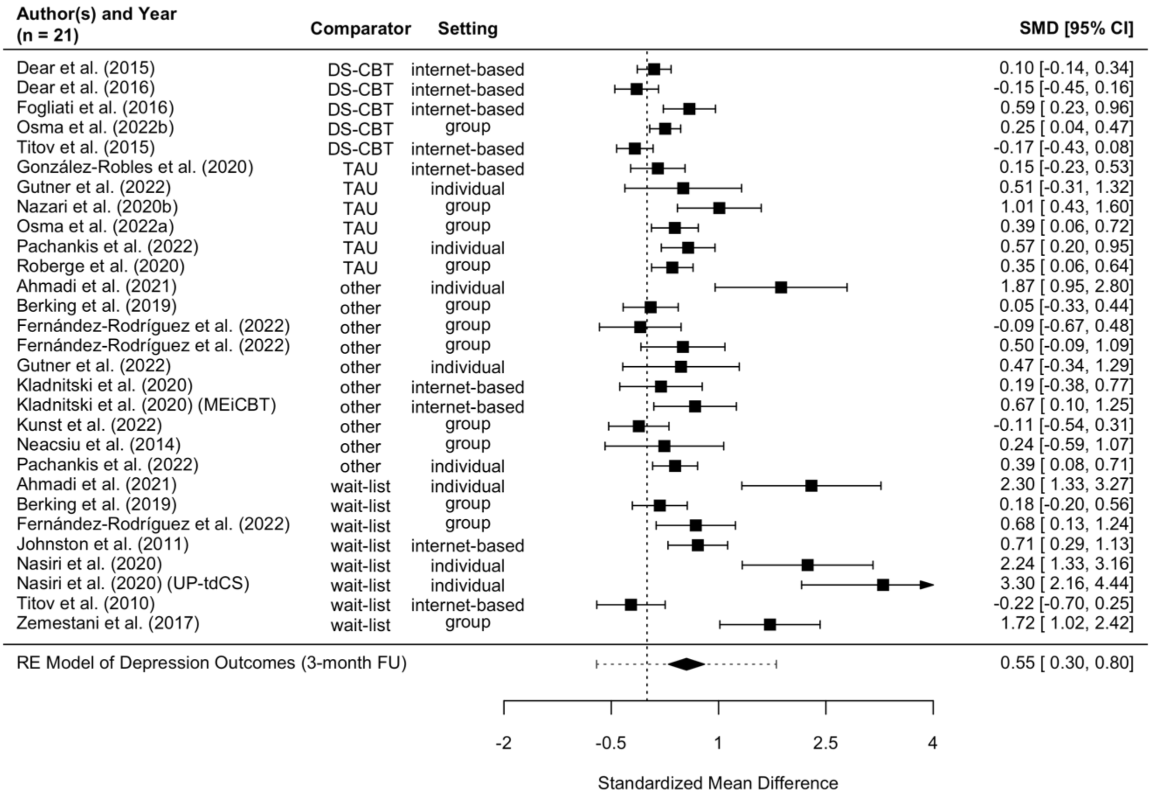

Supplementary Figure 2. Forest plot for depression comparing TD-CBT vs. controls at 3-month follow-up

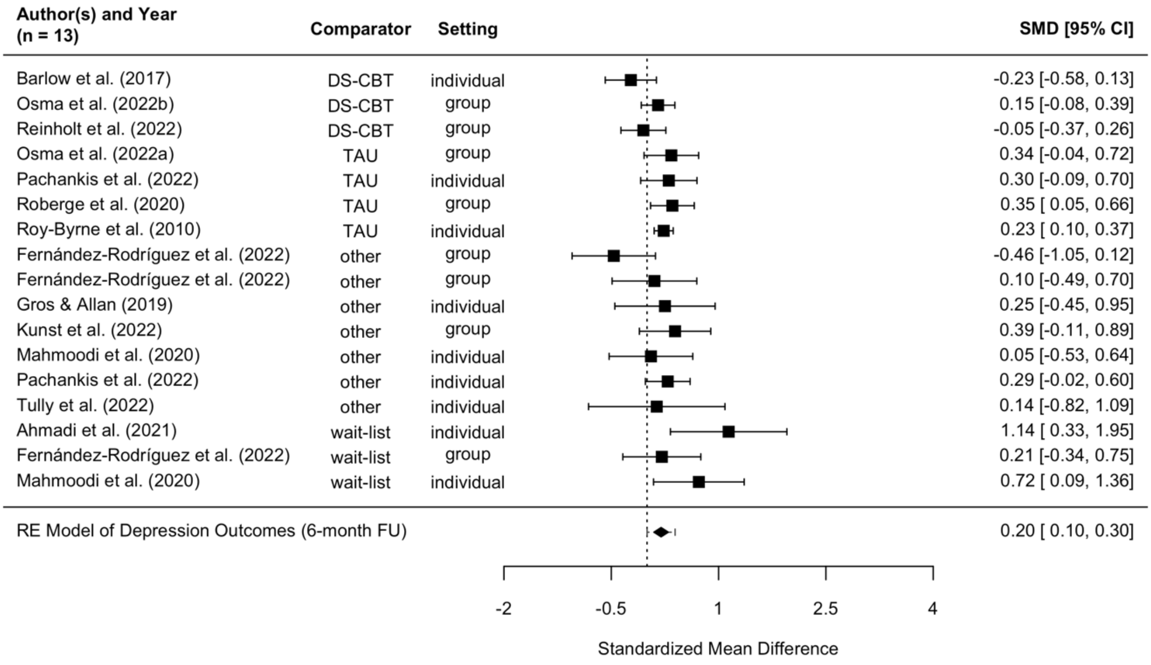

Supplementary Figure 3. Forest plot for depression comparing TD-CBT vs. controls at 6-month follow-up

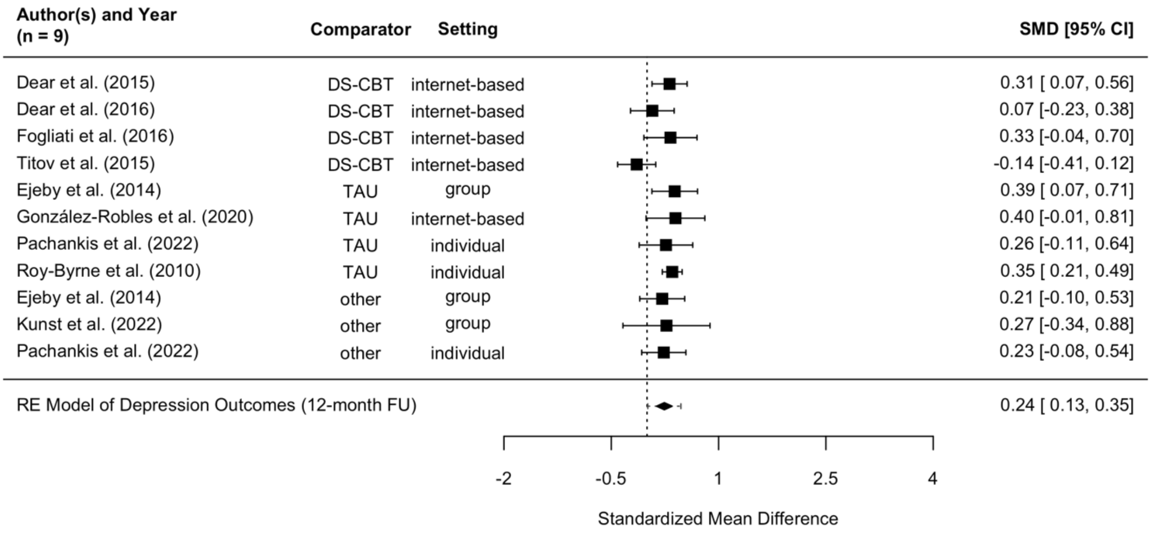

Supplementary Figure 4. Forest plot for depression comparing TD-CBT vs. controls at 12-month follow-up

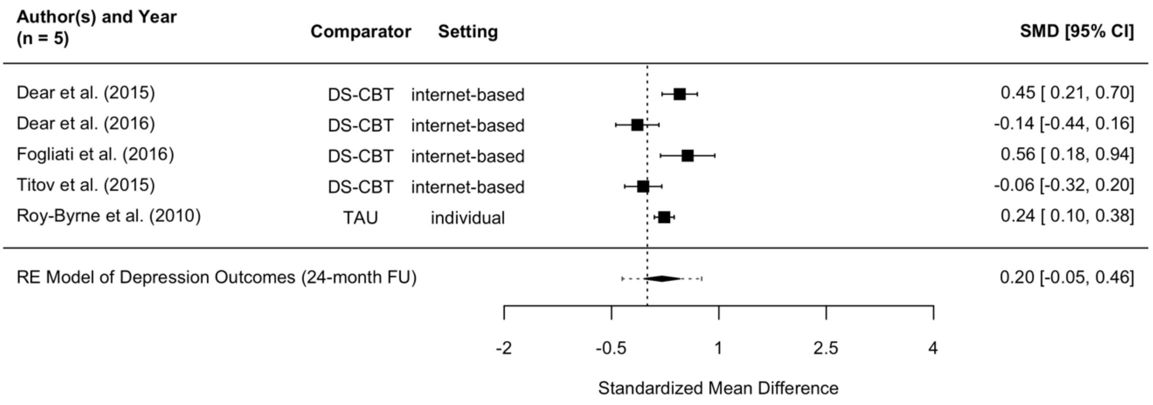

Supplementary Figure 5. Forest plot for depression comparing TD-CBT vs. controls at 24-month follow-up

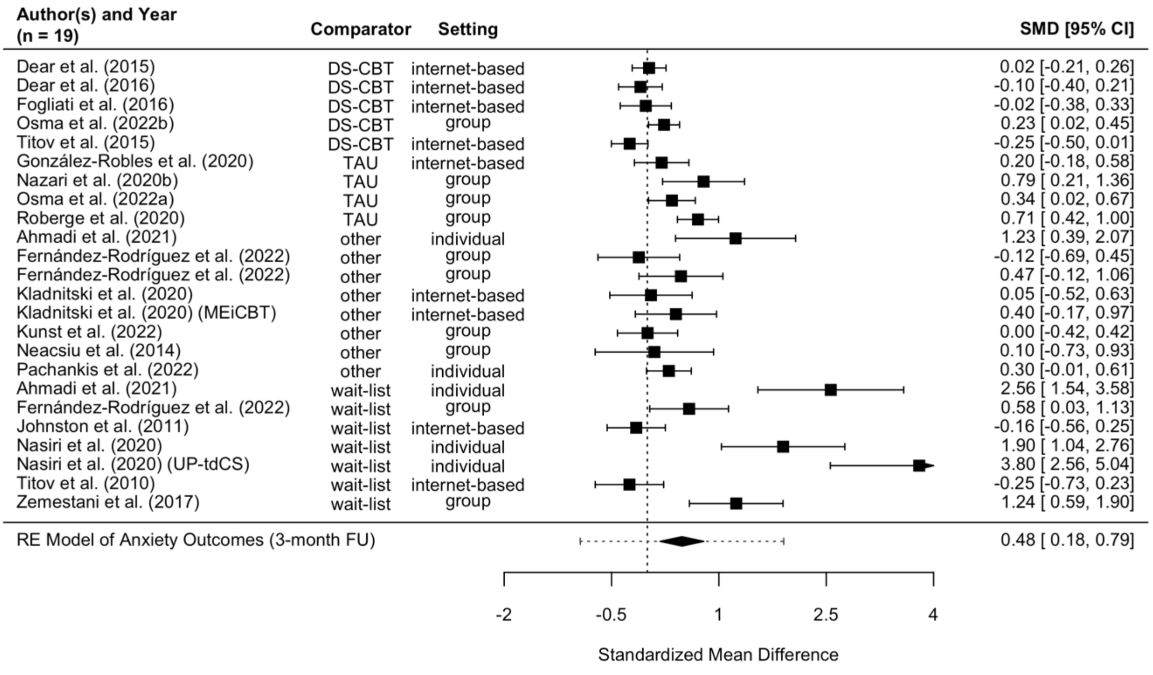

Supplementary Figure 6. Forest plot for anxiety comparing TD-CBT vs. controls at 3-month follow-up

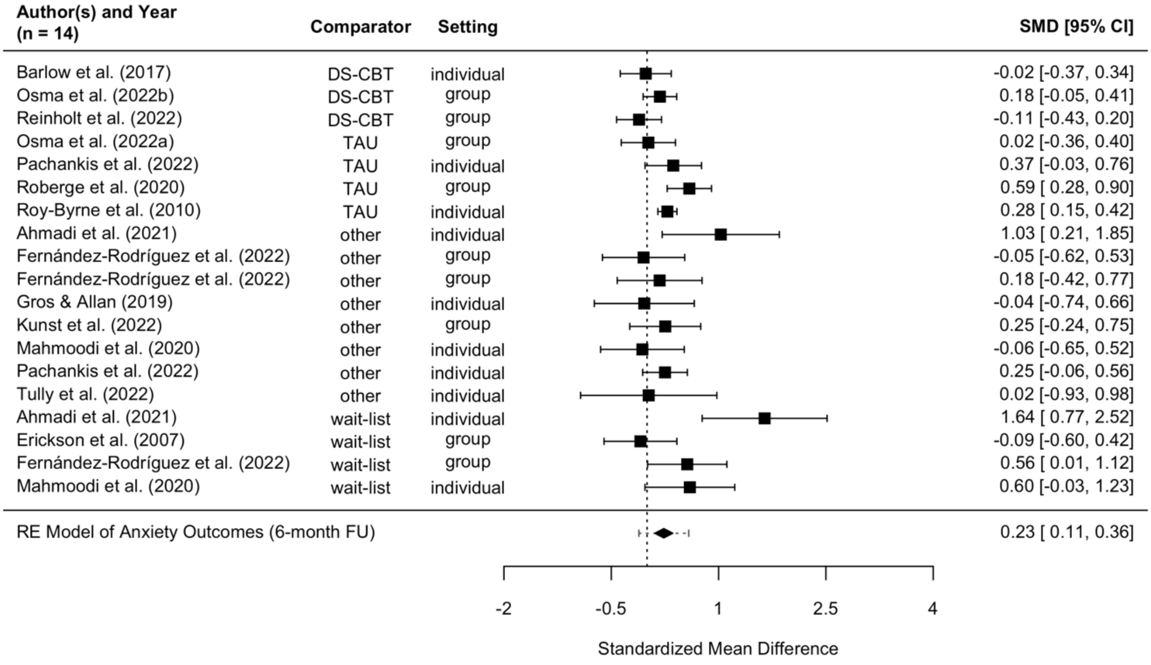

Supplementary Figure 7. Forest plot for anxiety comparing TD-CBT vs. controls at 6-month follow-up

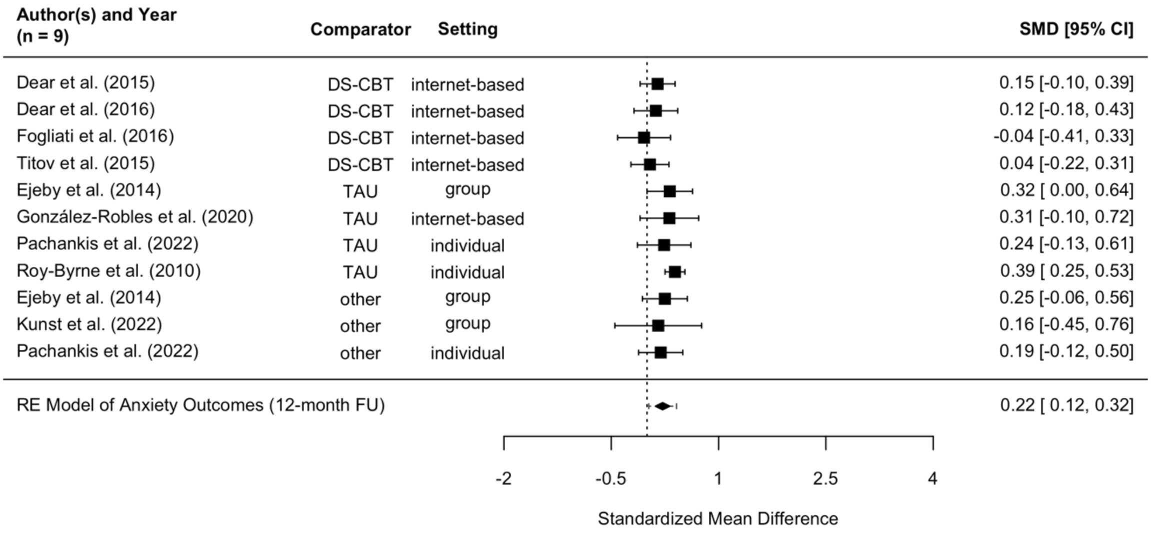

Supplementary Figure 8. Forest plot for anxiety comparing TD-CBT vs. controls at 12-month follow-up

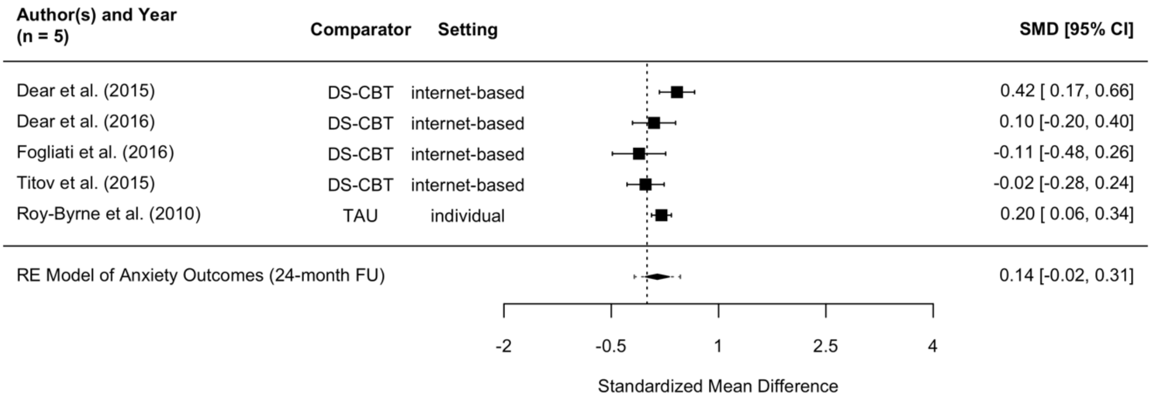

Supplementary Figure 9. Forest plot for anxiety comparing TD-CBT vs. controls at 24-month follow-up

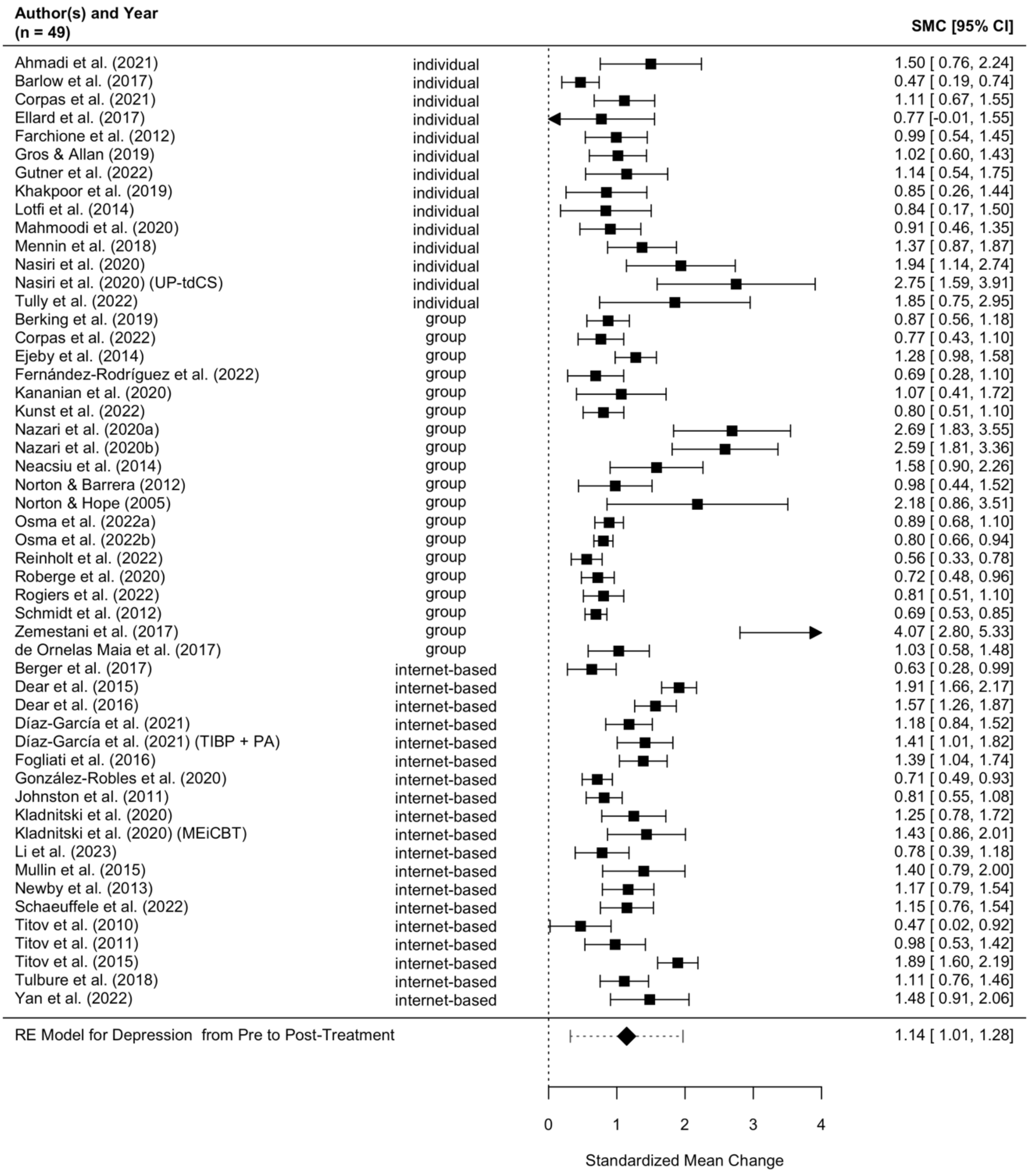

Supplementary Figure 10. Forest plot for depression from pre- to post-treatment

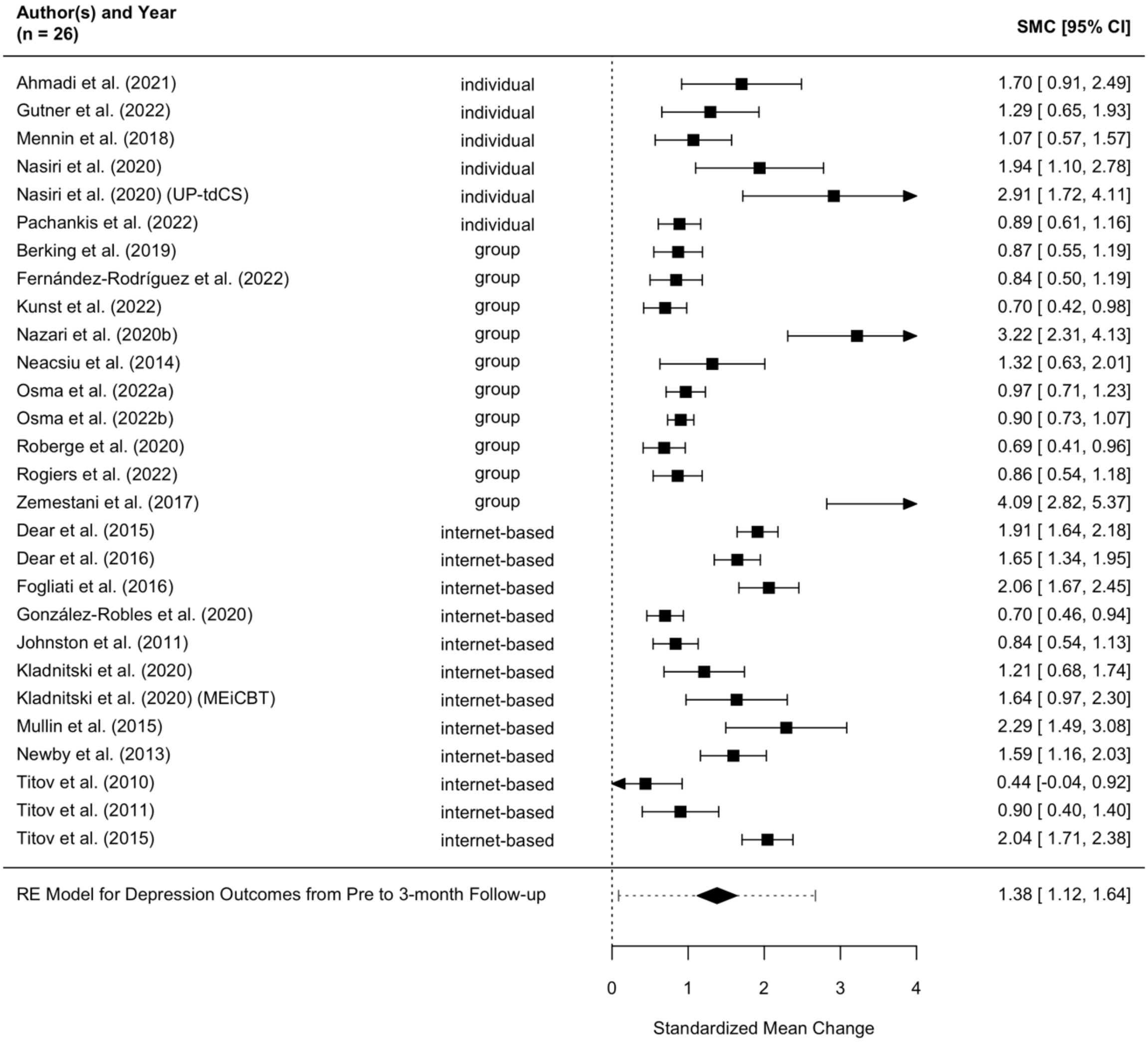

Supplementary Figure 11. Forest plot for depression from pre-treatment to 3-month follow-up

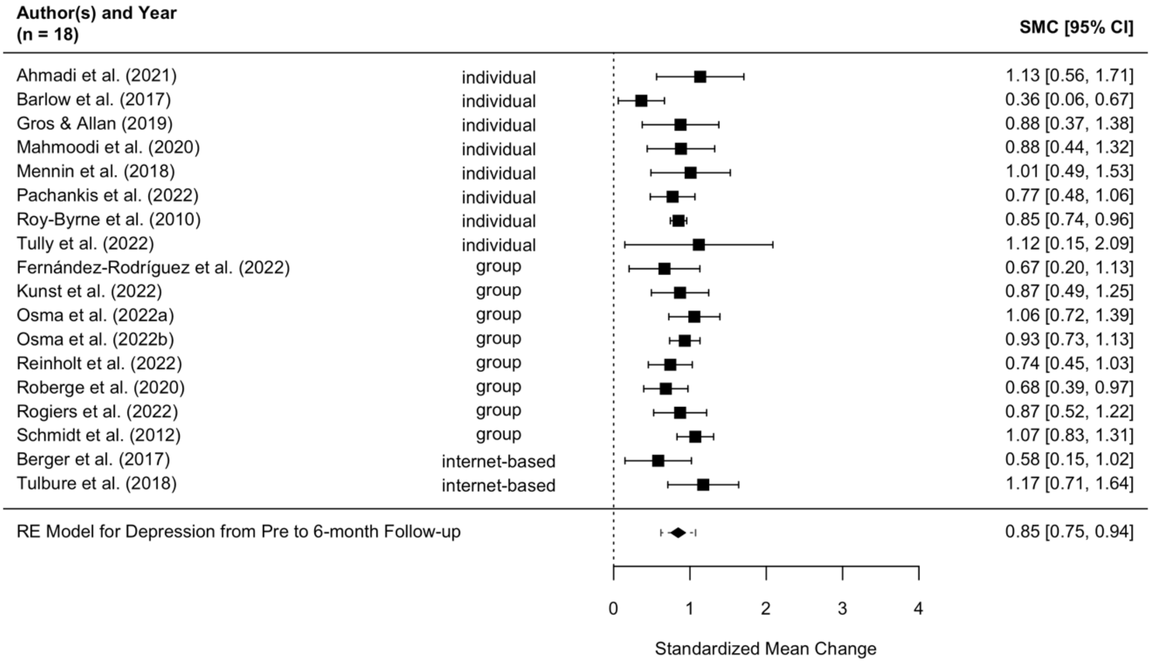

Supplementary Figure 12. Forest plot for depression from pre-treatment to 6-month follow-up

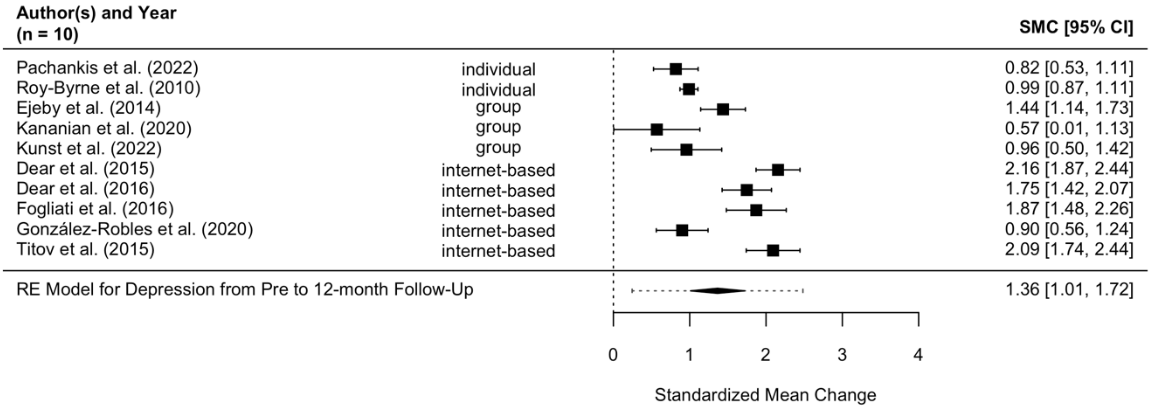

Supplementary Figure 13. Forest plot for depression from pre-treatment to 12-month follow-up

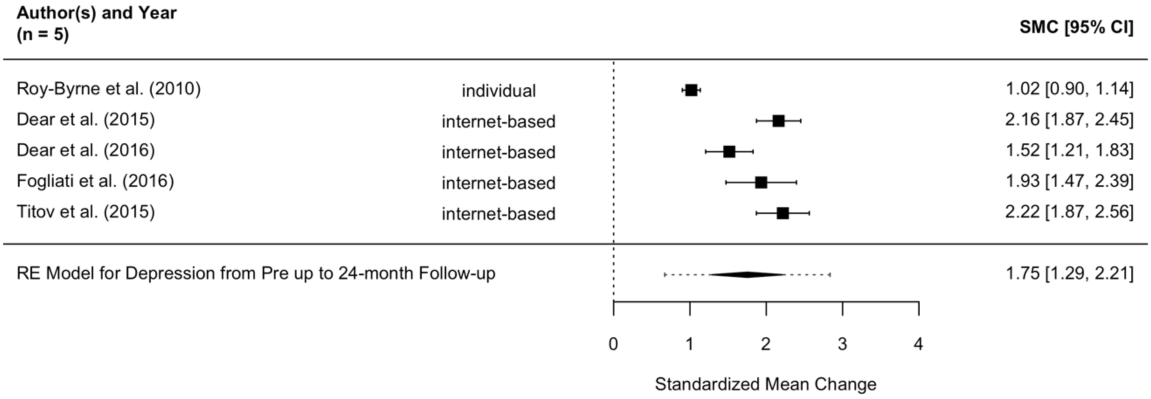

Supplementary Figure 14. Forest plot for depression from pre-treatment up to 24-month follow-up

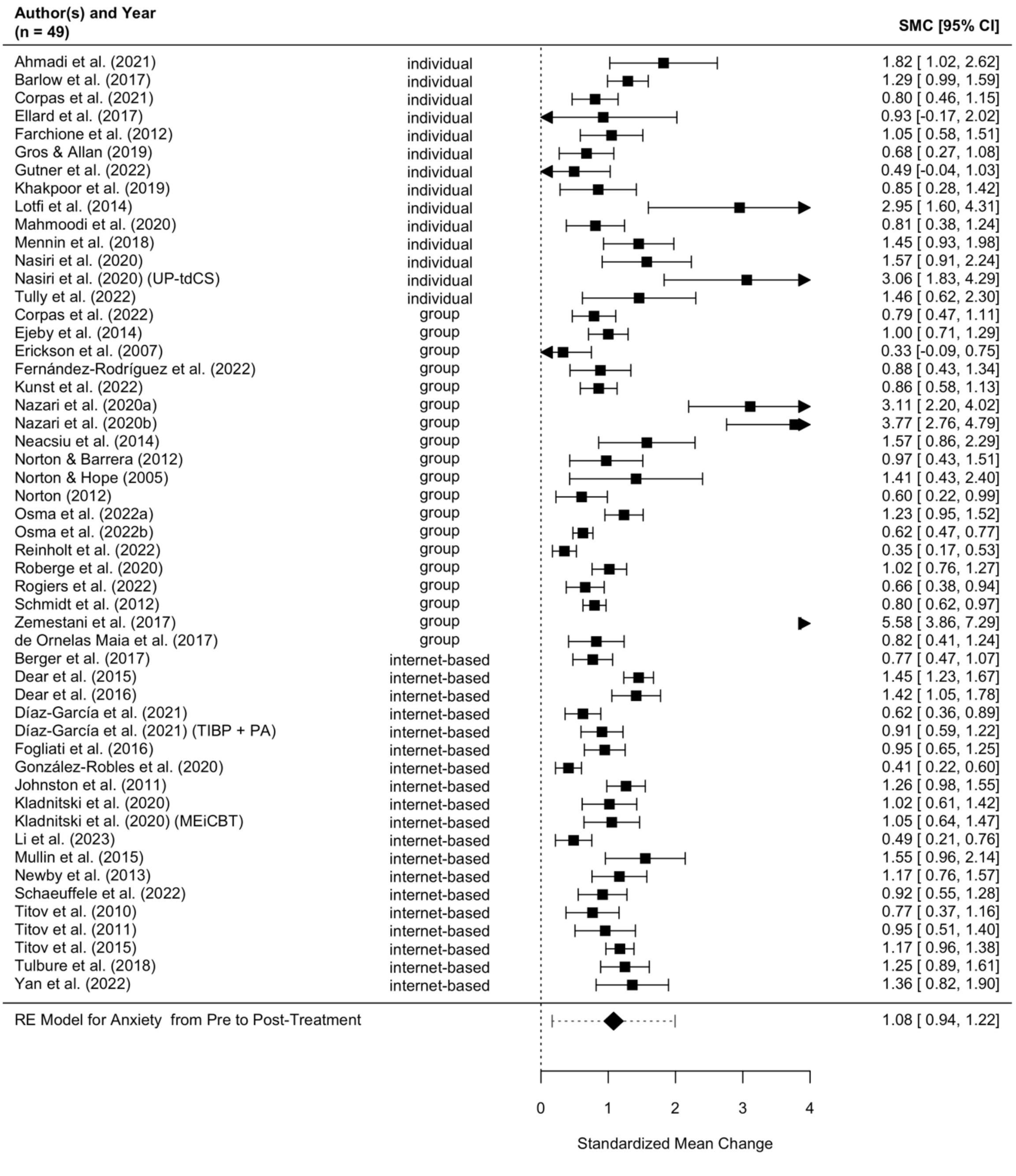

Supplementary Figure 15. Forest plot for anxiety from pre- to post-treatment

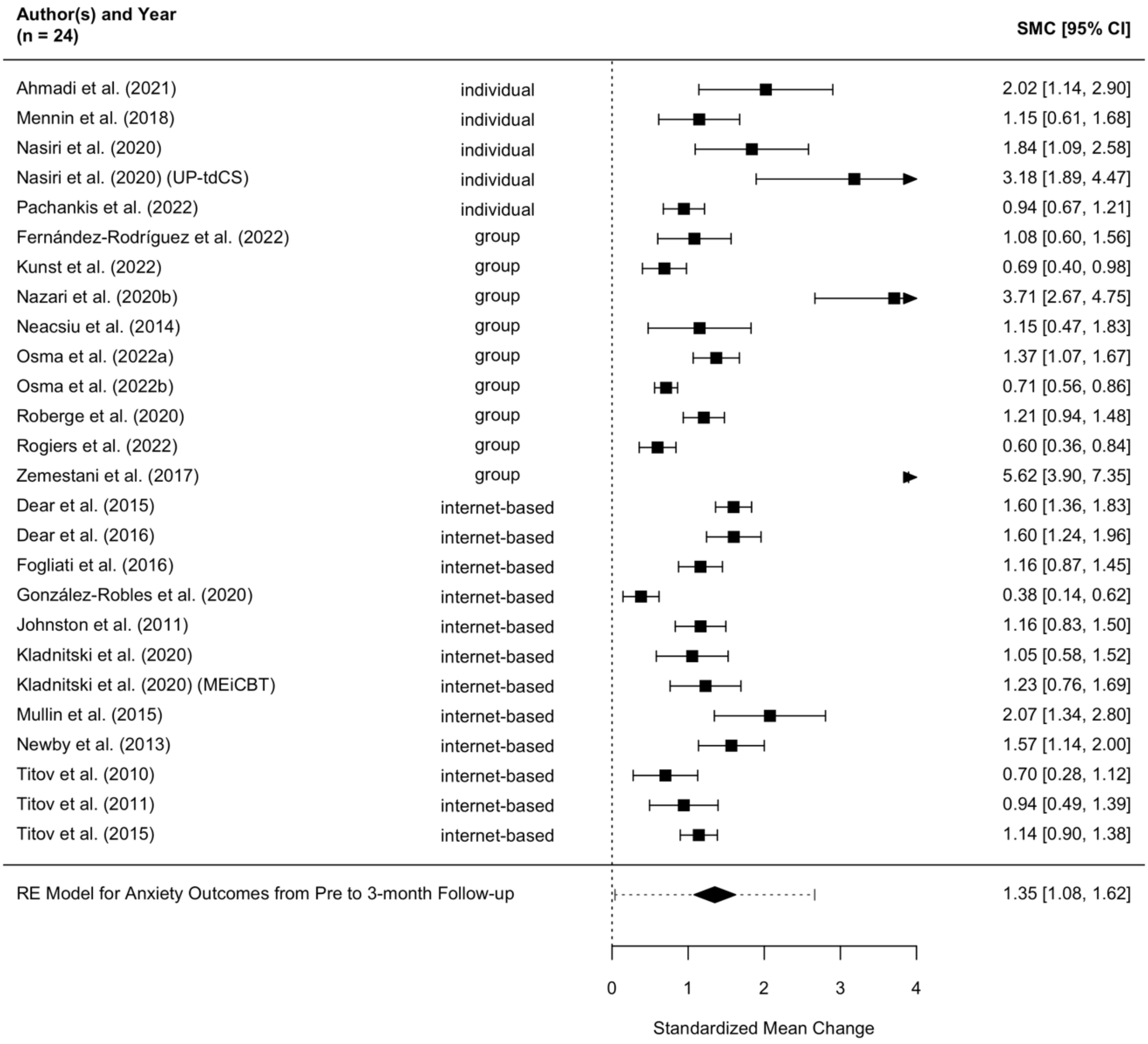

Supplementary Figure 16. Forest plot for anxiety from pre-treatment to 3-month follow-up

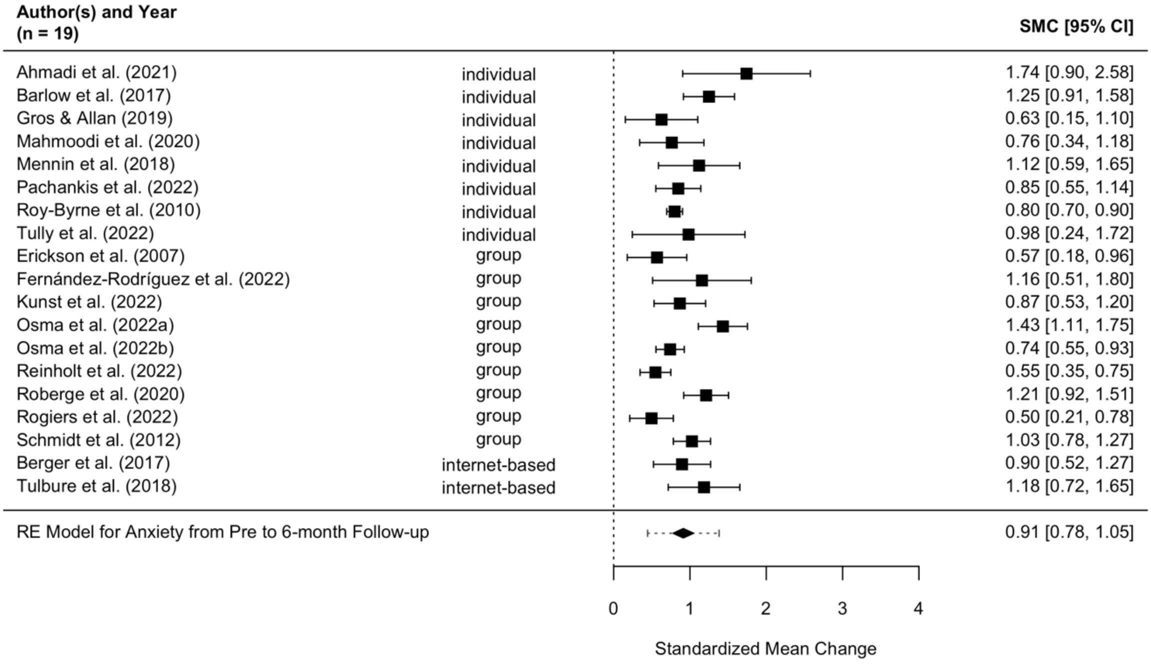

Supplementary Figure 17. Forest plot for anxiety from pre-treatment to 6-month follow-up

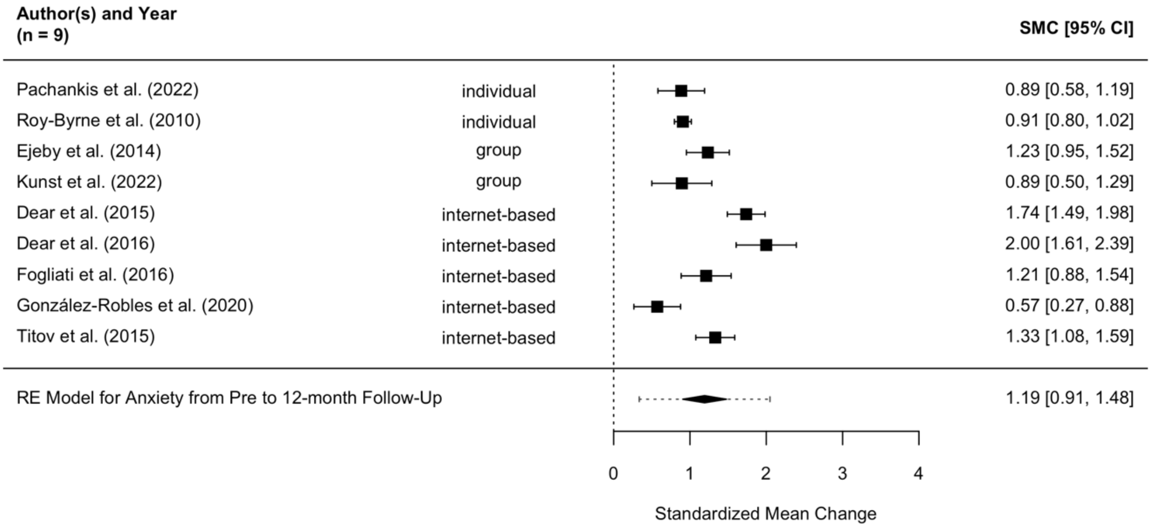

Supplementary Figure 18. Forest plot for anxiety from pre-treatment to 12-month follow-up

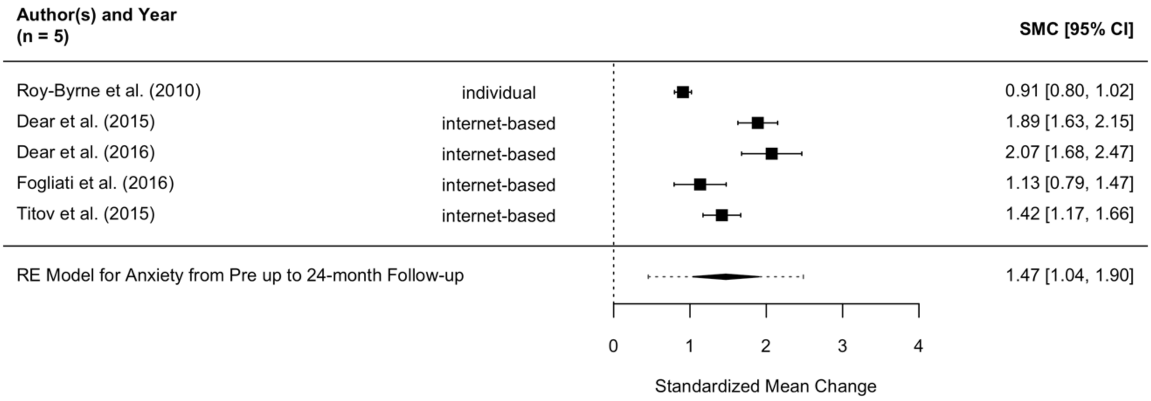

Supplementary Figure 19. Forest plot for anxiety from pre-treatment up to 24-month follow-up

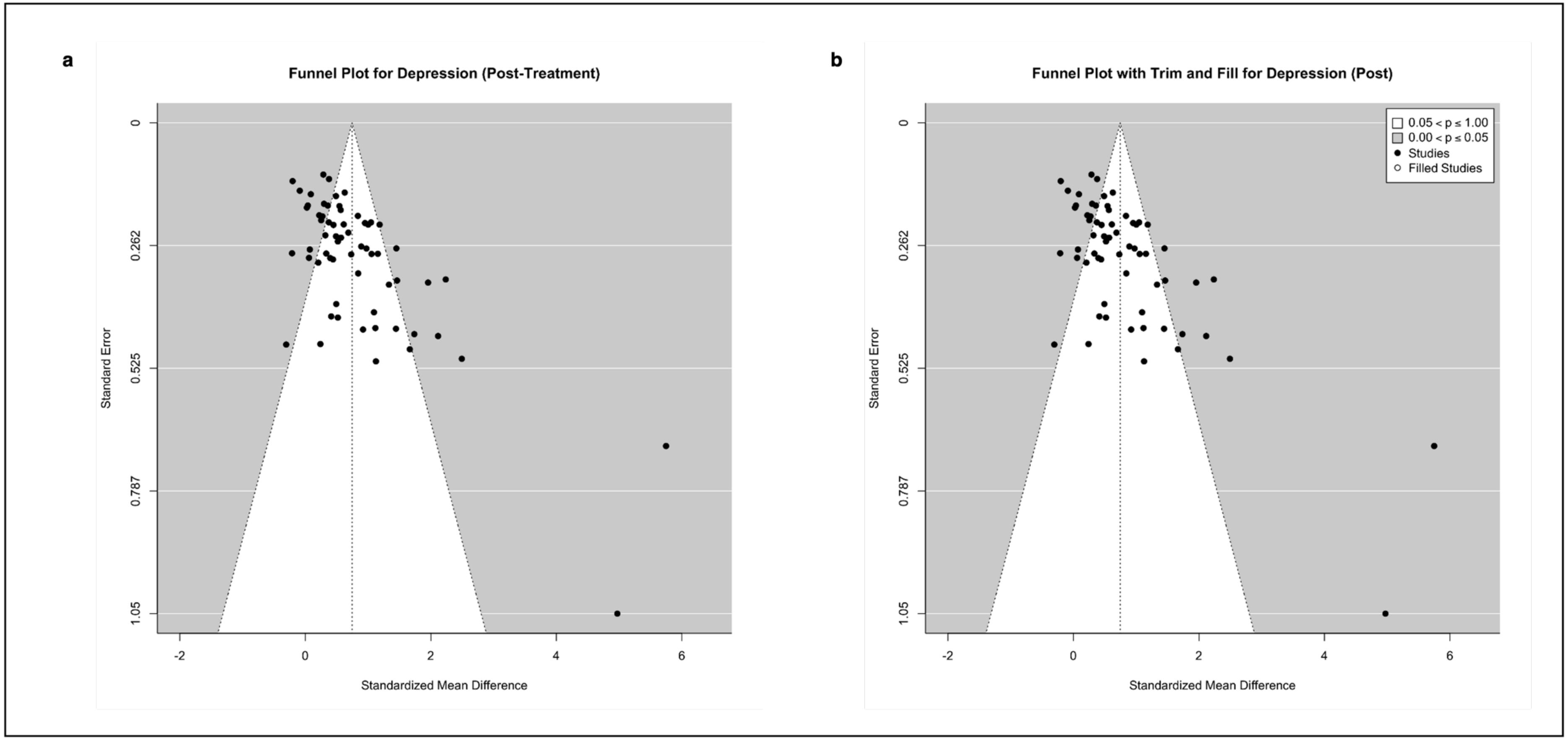

Supplementary Figure 20. Controlled Effects: Funnel plots for depression, a) without and b) with Trim and Fill Method

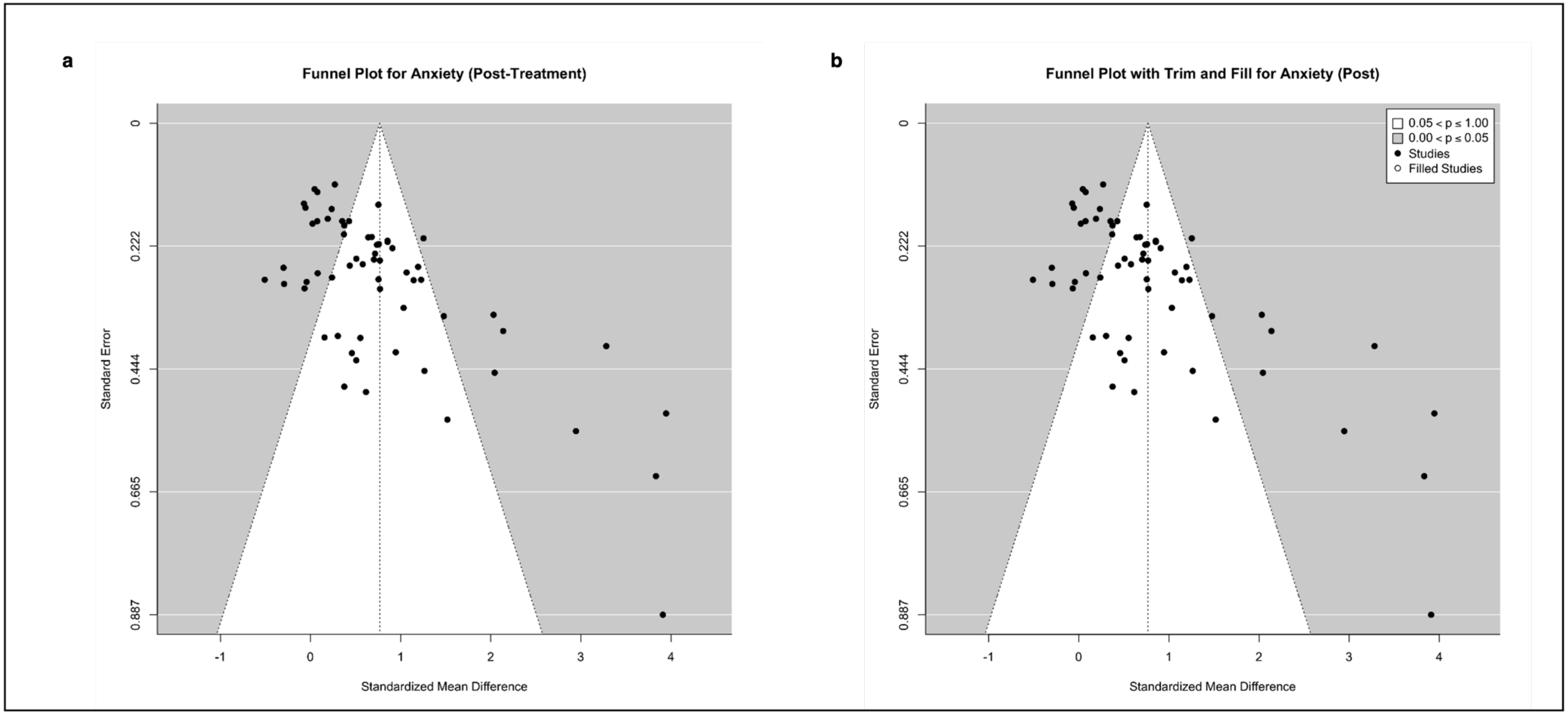

Supplementary Figure 21. Controlled Effects: Funnel plots for anxiety, a) without and b) with Trim and Fill Method

Additional Bayesian Analyses

1. Statistical Procedure

We corroborated the comparison between TD-CBT and DS-CBT for anxiety and depression post-treatment with additional Bayesian analyses. Using the brms-package, we applied a random-effects model and ran the analyses with 4000 iterations. Weakly informative priors were selected based on previous meta-analytic research (Harrer et al., 2021). For the distribution, we chose a normal distribution with a mean of 0 and a standard deviation of 1. This prior would imply that – in line with findings of meta-analytic comparisons of two active treatments - we expect approx. 95% of the effect size distribution to be values between -2 and 2. For the variance parameter, we chose a Half-Cauchy prior and set it to 0.5 (Williams et al., 2018). Half-Cauchy distributions are heavy tailed which makes less extreme values more likely. To assess the sensitivity of our analysis to different priors, we conducted a sensitivity analysis using alternative prior distributions. We considered a non-informative uniform prior as well as a stronger weakly informative prior to evaluate how different assumptions impact our results. Analysis code is shared on OSF.

1.1 Forest Plots of Comparisons of TD-CBT and DS-CBT

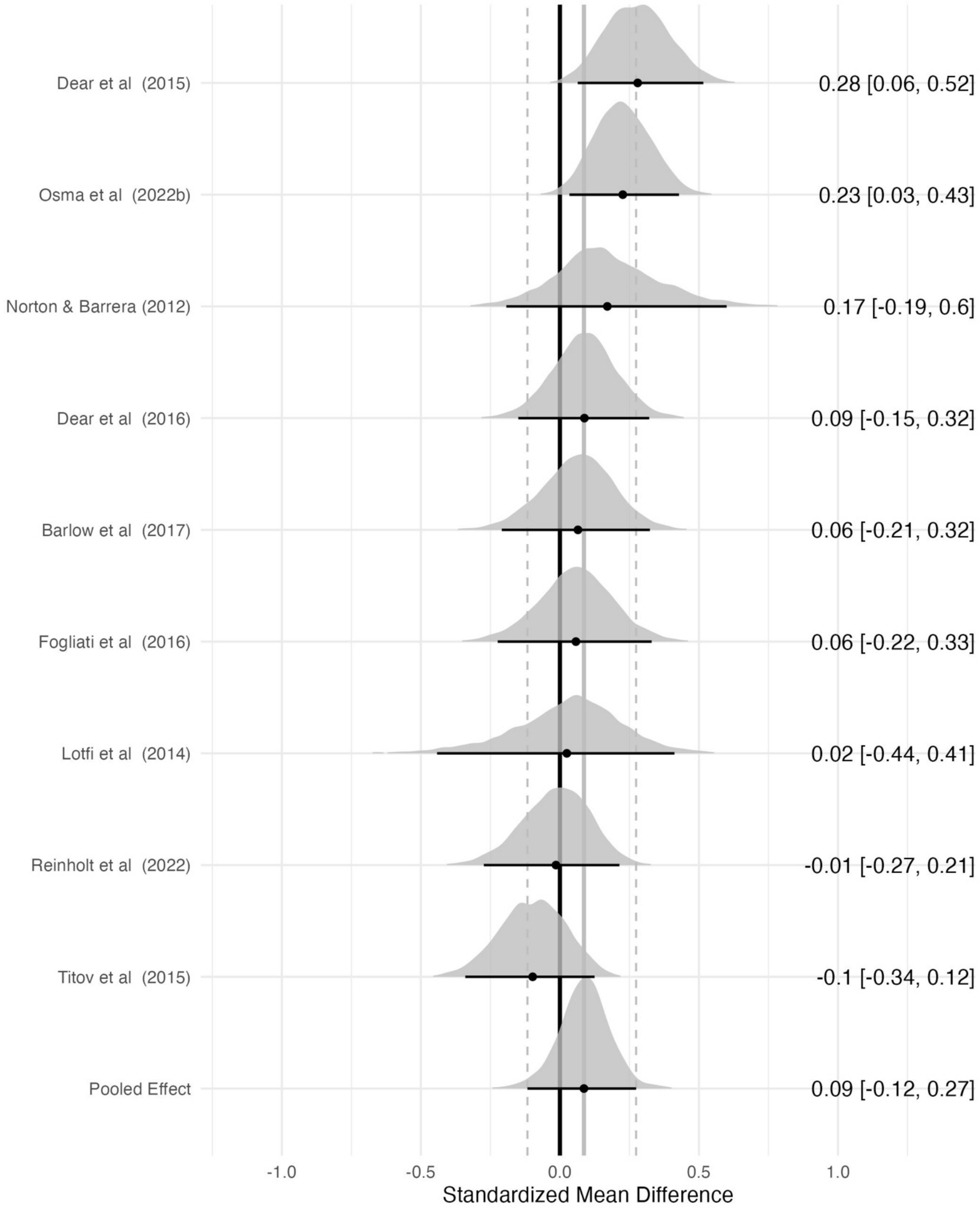

Supplementary Figure 22. Forest plot for depression comparing TD-CBT and DS-CBT (Bayesian analyses)

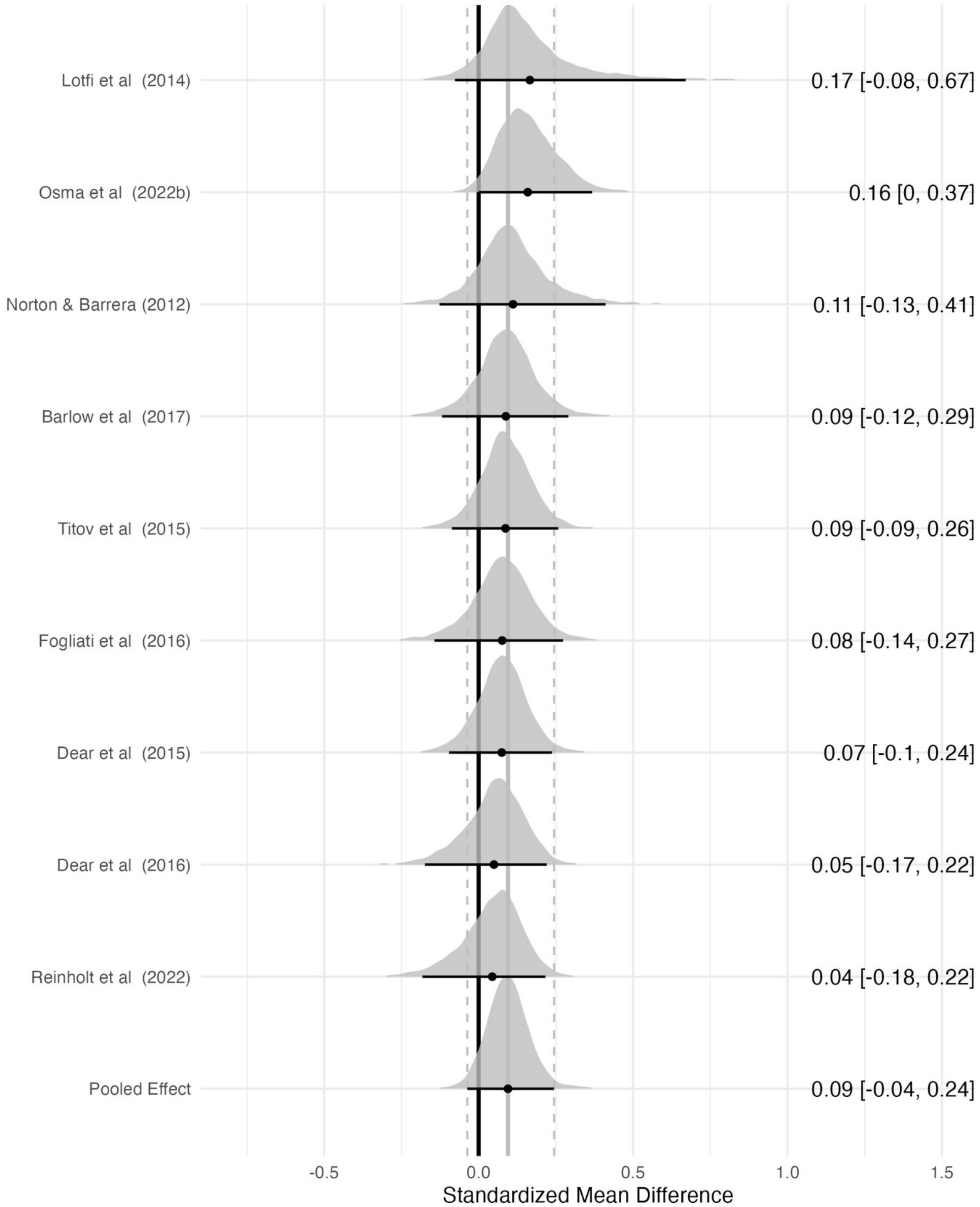

Supplementary Figure 23. Forest plot for anxiety comparing TD-CBT and DS-CBT (Bayesian analyses)

1.2 Sensitivity Analyses with Different Priors of Comparisons TD-CBT and DS-CBT

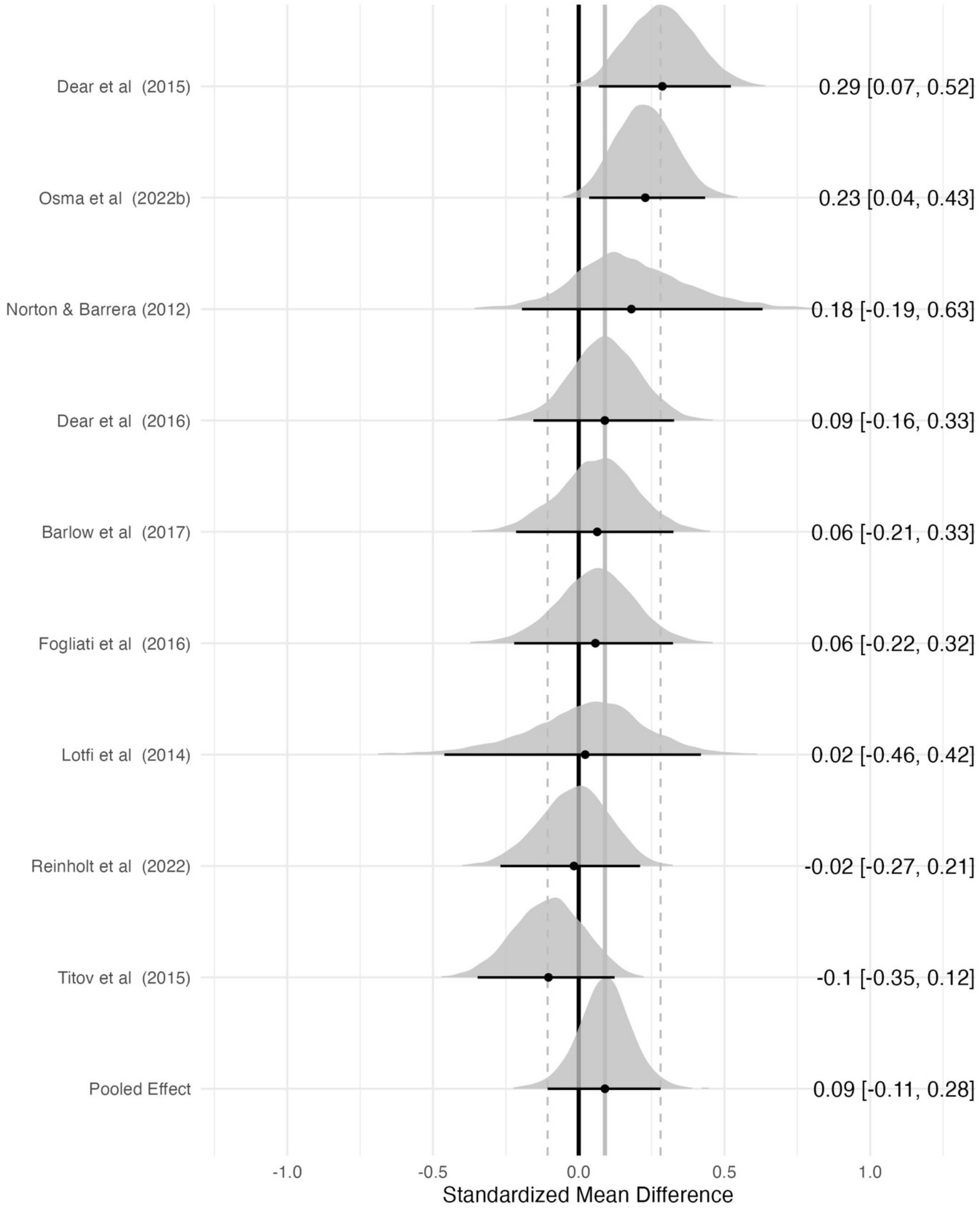

Supplementary Figure 24. Forest plot for depression comparing TD-CBT and DS-CBT applying a non-informative uniform prior with a wide range and a Cauchy prior with a large scale

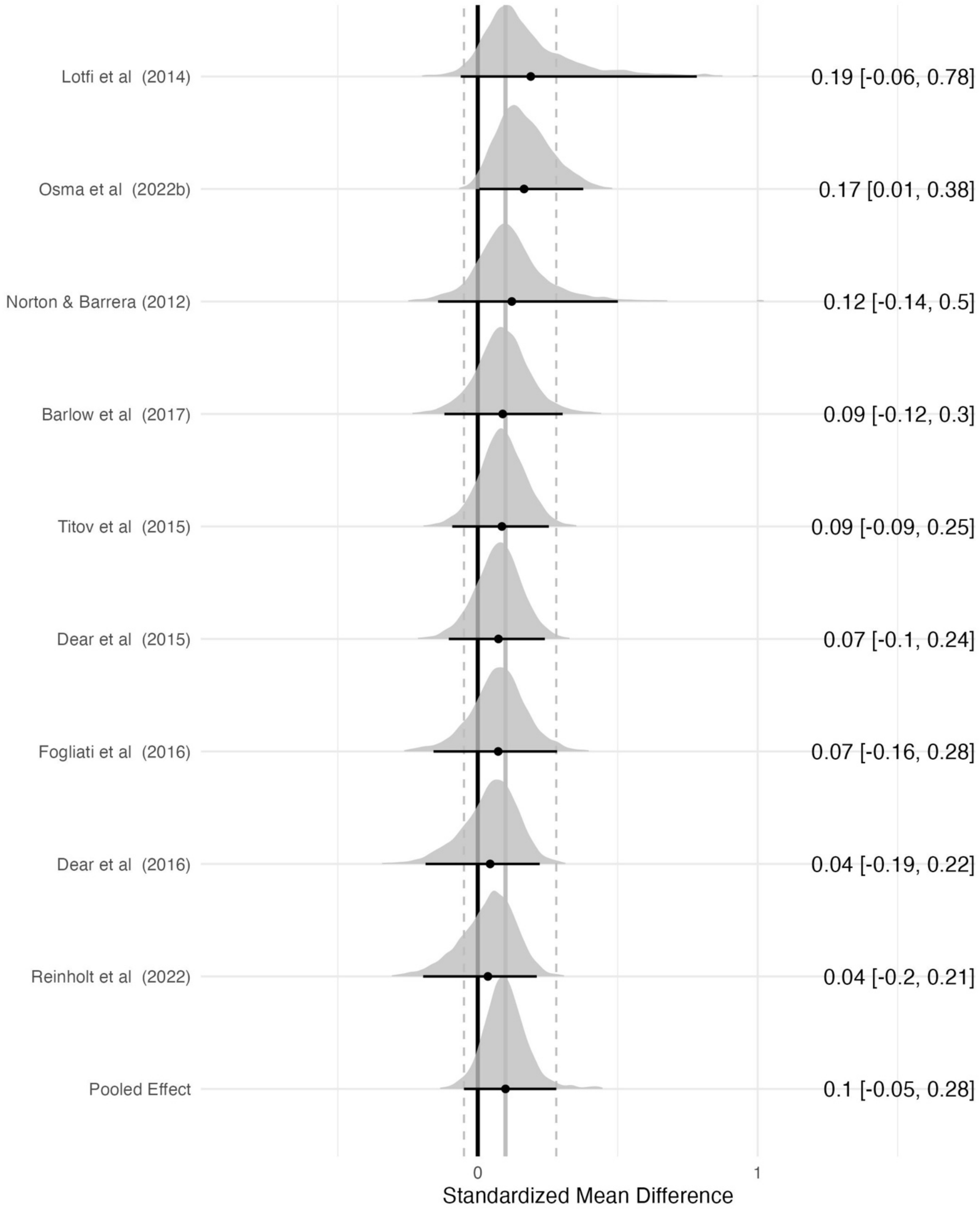

Supplementary Figure 25. Forest plot for anxiety comparing TD-CBT and DS-CBT applying a non-informative uniform prior with a wide range and a Cauchy prior with a large scale

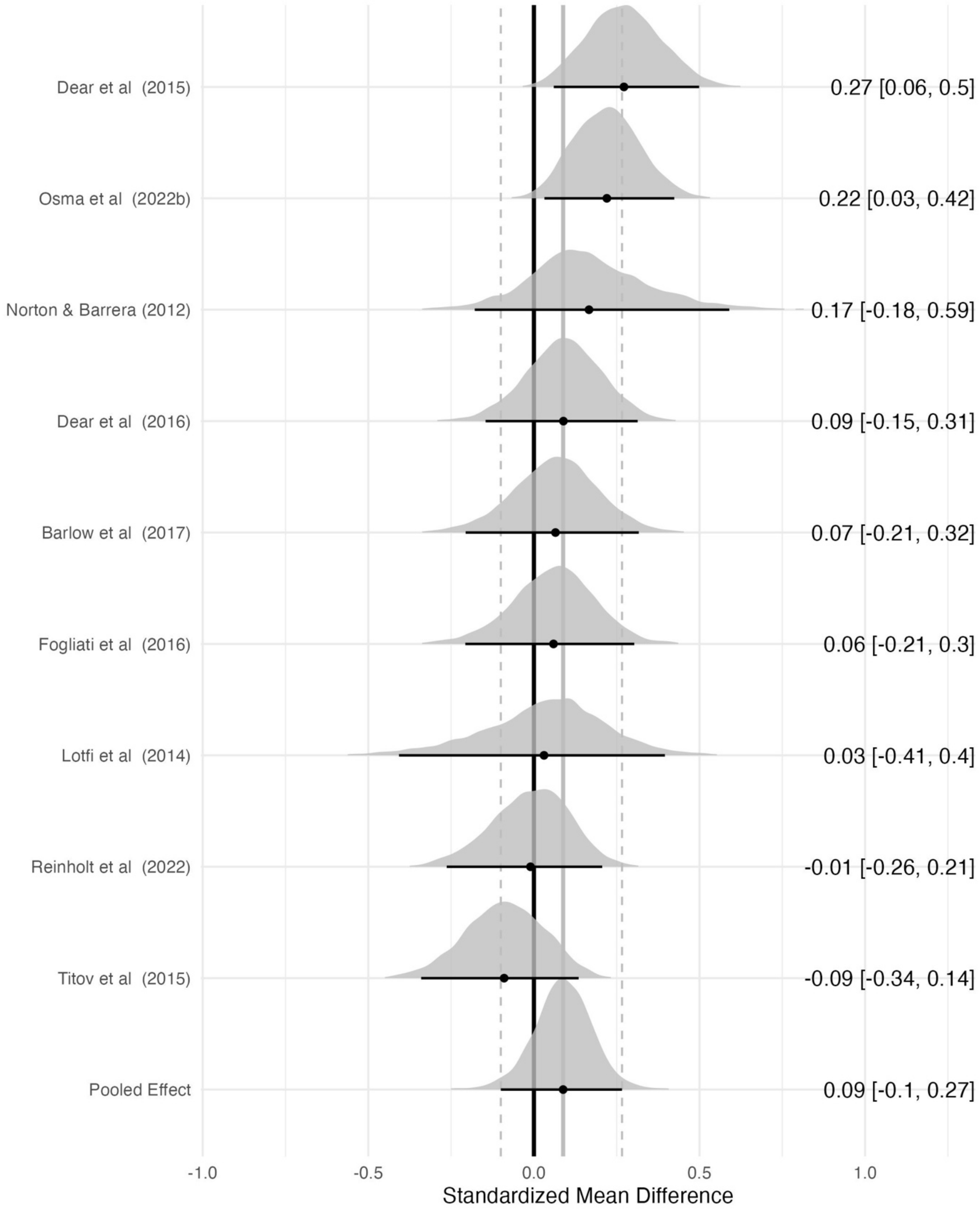

Supplementary Figure 26. Forest plot for depression comparing TD-CBT and DS-CBT applying a weakly informative prior with a smaller distribution and a less conservative Half-Cauchy prior

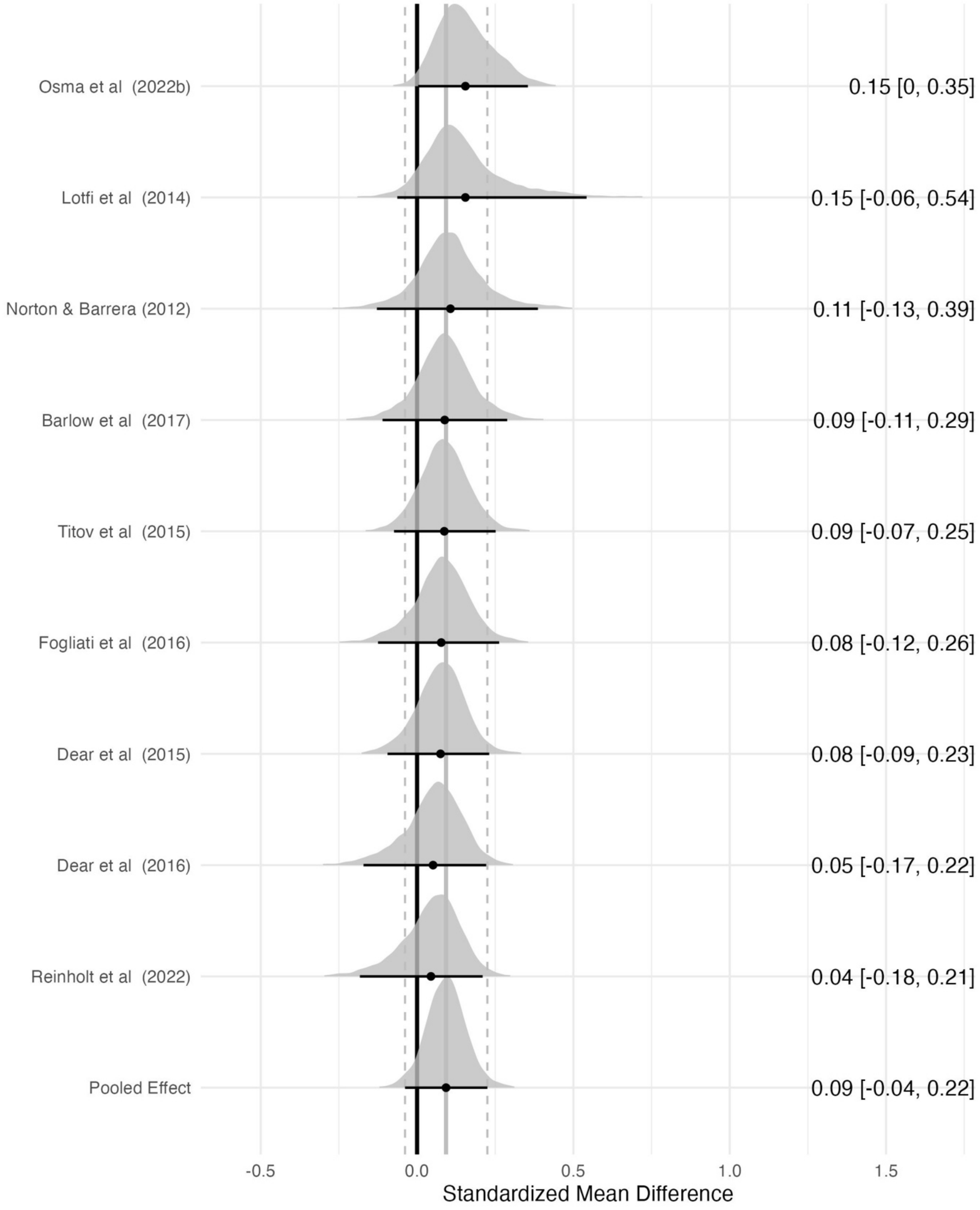

Supplementary Figure 27. Forest plot for anxiety comparing TD-CBT and DS-CBT applying a weakly informative prior with a smaller distribution and a less conservative Half-Cauchy prior
