# Supplementary material for: A systematic review and meta-analysis of transdiagnostic cognitive behavioural therapies for emotional disorders
Source: Nat Hum Behav. 2024 Jan 16;8(3):493–509. doi: 10.1038/s41562-023-01787-3 (PMC10963275; doi:10.1038/s41562-023-01787-3)
Supplement: Supplementary file 2 — Reporting Summary [file 41562_2023_1787_MOESM2_ESM.pdf]

nature portfolio

Corresponding author(s): Laura Meine

Last updated by author(s): Oct 2, 2023

Reporting Summary

Nature Portfolio wishes to improve the reproducibility of the work that we publish. This form provides structure for consistency and transparency in reporting. For further information on Nature Portfolio policies, see our Editorial Policies and the Editorial Policy Checklist.

Statistics

For all statistical analyses, confirm that the following items are present in the figure legend, table legend, main text, or Methods section.

n/a	Confirmed	
☐	☒	The exact sample size (n) for each experimental group/condition, given as a discrete number and unit of measurement
☒	☐	A statement on whether measurements were taken from distinct samples or whether the same sample was measured repeatedly
☐	☒	The statistical test(s) used AND whether they are one- or two-sided<br>*Only common tests should be described solely by name; describe more complex techniques in the Methods section.*
☒	☐	A description of all covariates tested
☐	☒	A description of any assumptions or corrections, such as tests of normality and adjustment for multiple comparisons
☐	☒	A full description of the statistical parameters including central tendency (e.g. means) or other basic estimates (e.g. regression coefficient) AND variation (e.g. standard deviation) or associated estimates of uncertainty (e.g. confidence intervals)
☐	☒	For null hypothesis testing, the test statistic (e.g. F, t, r) with confidence intervals, effect sizes, degrees of freedom and P value noted<br>*Give P values as exact values whenever suitable.*
☒	☐	For Bayesian analysis, information on the choice of priors and Markov chain Monte Carlo settings
☒	☐	For hierarchical and complex designs, identification of the appropriate level for tests and full reporting of outcomes
☐	☒	Estimates of effect sizes (e.g. Cohen's d, Pearson's r), indicating how they were calculated

Our web collection on statistics for biologists contains articles on many of the points above.

Software and code

Policy information about availability of computer code

Data collection	Zotero (Version 6.0.23), Google Sheets
Data analysis	All analyses were conducted in R (version 4.3.1), using the metafor (Viechtbauer, 2010; version 4.2-0), meta (Balduzzi et al., 2019; version 6.5-0), and dmetar package (Harrer et al., 2019; version 0.1.0). Risk of bias was evaluated with the Cochrane risk-of-bias tool (RoB 2.0). All code is available on the Open Science Framework (OSF) at https://osf.io/ta4fg.

For manuscripts utilizing custom algorithms or software that are central to the research but not yet described in published literature, software must be made available to editors and reviewers. We strongly encourage code deposition in a community repository (e.g. GitHub). See the Nature Portfolio guidelines for submitting code & software for further information.

Data

Policy information about availability of data

All manuscripts must include a data availability statement. This statement should provide the following information, where applicable:

- Accession codes, unique identifiers, or web links for publicly available datasets
- A description of any restrictions on data availability
- For clinical datasets or third party data, please ensure that the statement adheres to our policy

All data for this project, along with relevant code and codebooks, are publicly available on the OSF at https://osf.io/ta4fg.

Research involving human participants, their data, or biological material

Policy information about studies with human participants or human data. See also policy information about sex, gender (identity/presentation), and sexual orientation and race, ethnicity and racism.

Reporting on sex and gender	*Use the terms sex (biological attribute) and gender (shaped by social and cultural circumstances) carefully in order to avoid confusing both terms. Indicate if findings apply to only one sex or gender; describe whether sex and gender were considered in study design; whether sex and/or gender was determined based on self-reporting or assigned and methods used.*<br>*Provide in the source data disaggregated sex and gender data, where this information has been collected, and if consent has been obtained for sharing of individual-level data; provide overall numbers in this Reporting Summary. Please state if this information has not been collected.*<br>*Report sex- and gender-based analyses where performed, justify reasons for lack of sex- and gender-based analysis.*
Reporting on race, ethnicity, or other socially relevant groupings	*Please specify the socially constructed or socially relevant categorization variable(s) used in your manuscript and explain why they were used. Please note that such variables should not be used as proxies for other socially constructed/relevant variables (for example, race/ethnicity should not be used as a proxy for socioeconomic status).*<br>*Provide clear definitions of the relevant terms used, how they were provided (by the participants/respondents, the researchers, or third parties), and the method(s) used to classify people into the different categories (e.g. self-report, census or administrative data, social media data, etc.)*<br>*Please provide details about how you controlled for confounding variables in your analyses.*
Population characteristics	*Describe the covariate-relevant population characteristics of the human research participants (e.g. age, genotypic information, past and current diagnosis and treatment categories). If you filled out the behavioural & social sciences study design questions and have nothing to add here, write "See above."*
Recruitment	*Describe how participants were recruited. Outline any potential self-selection bias or other biases that may be present and how these are likely to impact results.*
Ethics oversight	*Identify the organization(s) that approved the study protocol.*

Note that full information on the approval of the study protocol must also be provided in the manuscript.

Field-specific reporting

Please select the one below that is the best fit for your research. If you are not sure, read the appropriate sections before making your selection.

☐ Life sciences ☒ Behavioural & social sciences ☐ Ecological, evolutionary & environmental sciences

For a reference copy of the document with all sections, see nature.com/documents/nr-reporting-summary-flat.pdf

Behavioural & social sciences study design

All studies must disclose on these points even when the disclosure is negative.

Study description	Systematic review and meta-analysis with quantitative analysis of sub-groups
Research sample	Randomised controlled trials evaluating transdiagnostic, CBT-based psychotherapy treatments (TD-CBT) in adult patients diagnosed with an emotional disorder. We included studies published from January 2000 that investigated TD-CBT in individual, group, or Internet-based setting compared to a wait-list control condition, treatment-as-usual, disorder-specific treatment or another active psychological intervention. Studies needed to include a self-report measure of anxiety and/or depression severity at pre- and post-treatment. The dataset compiled from extracted data of included studies is publicly available on the OSF.
Sampling strategy	We formulated clear inclusion and exclusion criteria and then conducted a systematic literature search on PubMed and MEDLINE, PsycINFO, Google Scholar, medRxiv (incl. bioRxiv), and OSF Preprints up to 12/01/2023. All studies that met the inclusion criteria and did not fulfill any exclusion criterion were included for analyses.
Data collection	Articles found through the above mentioned systematic search that matched our inclusion criteria were collected in Zotero. Data for the meta-analysis was extracted by two independent reviewers and saved in a Google sheet (see available data). As this was a systematic review and meta-analysis, the researchers were not blinded.
Timing	Studies published between January 2000 and 12/01/2023 were included.
Data exclusions	For the meta-analytic calculations, we excluded one study because the data were not available and another two studies because no self-report of anxiety or depression was available.
Non-participation	129 studies were full-text screened but met at least one exclusion criterion and were therefore not considered for analyses. Reasons for exclusions were: secondary analysis (n = 18), no clinician-established diagnosis (n = 35), different primary diagnosis (n = 4), not based on transdiagnostic CBT principles (n = 19), tailored treatment (n = 8), anxiety/depression outcome not available (n = 2), no RCT (n = 19), study protocol (n = 8), meta-analysis (n = 2), factual text (n = 2), other (n = 12).

Randomization	Only RCTs were included in this review and meta-analysis.

Reporting for specific materials, systems and methods

We require information from authors about some types of materials, experimental systems and methods used in many studies. Here, indicate whether each material, system or method listed is relevant to your study. If you are not sure if a list item applies to your research, read the appropriate section before selecting a response.

Materials & experimental systems

n/a	Involved in the study
☒	☐ Antibodies
☒	☐ Eukaryotic cell lines
☒	☐ Palaeontology and archaeology
☒	☐ Animals and other organisms
☒	☐ Clinical data
☒	☐ Dual use research of concern
☒	☐ Plants

Methods

n/a	Involved in the study
☒	☐ ChIP-seq
☒	☐ Flow cytometry
☒	☐ MRI-based neuroimaging
